# Lysosomal Trafficking, Antigen Presentation, and Microbial Killing Are Controlled by the Arf-like GTPase Arl8b

**DOI:** 10.1016/j.immuni.2011.06.009

**Published:** 2011-08-26

**Authors:** Salil Garg, Mahak Sharma, Cindy Ung, Amit Tuli, Duarte C. Barral, David L. Hava, Natacha Veerapen, Gurdyal S. Besra, Nir Hacohen, Michael B. Brenner

**Affiliations:** 1 Harvard Division of Medical Sciences, Graduate Program in Immunology and Harvard-MIT MD PhD Program, Boston, MA 02115, USA; 2 Division of Rheumatology, Immunology and Allergy, Department of Medicine, Brigham and Women's Hospital, Boston, MA 02115, USA; 3 School of Biosciences, University of Birmingham, Edgbaston, Birmingham, United Kingdom; 4 Broad Institute of MIT and Harvard, Cambridge, MA 02142, USA; 5 Center for Immunology and Inflammatory Diseases, Massachusetts General Hospital, Charlestown, MA 02129, USA

## Abstract

Antigen presentation and microbial killing are critical arms of host defense that depend upon cargo trafficking into lysosomes. Yet, the molecular regulators of traffic into lysosomes are only partly understood. Here, using a lysosome-dependent immunological screen of a trafficking shRNA library, we identified the Arf-like GTPase Arl8b as a critical regulator of cargo delivery to lysosomes. Homotypic fusion and vacuole protein sorting (HOPS) complex members were identified as effectors of Arl8b and were dependent on Arl8b for recruitment to lysosomes, suggesting that Arl8b-HOPS plays a general role in directing traffic to lysosomes. Moreover, the formation of CD1 antigen-presenting complexes in lysosomes, their delivery to the plasma membrane, and phagosome-lysosome fusion were all markedly impaired in Arl8b silenced cells resulting in corresponding defects in T cell activation and microbial killing. Together, these results define Arl8b as a key regulator of lysosomal cellular and immunological functions.

## Graphical abstract

**Graphical Abstract:**

**Figure:**
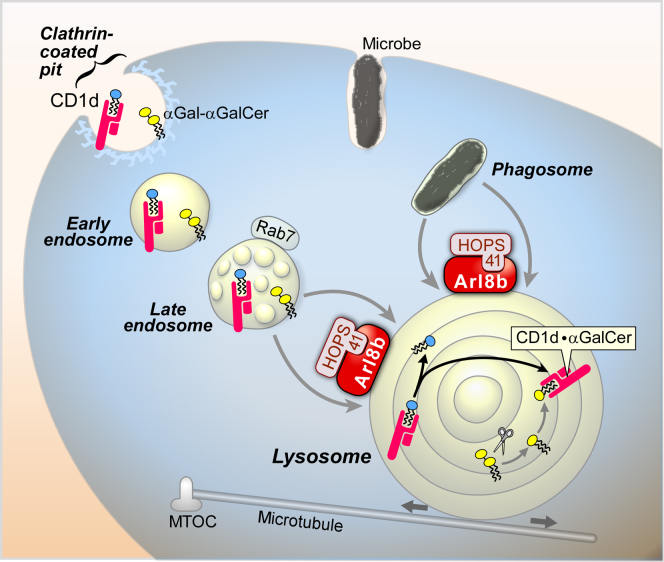

**Highlights:**

► Arl8b silencing reduces lysosomal CD1d antigen presentation to NKT-cells ► Arl8b controls trafficking of endocytosed dextran, LDL, and CD1d to lysosomes ► Arl8b binds VPS41 and recruits HOPS Complex members to lysosomes ► Arl8b controls phagosome to lysosome trafficking and microbial killing

## Introduction

Intracellular trafficking to and from lysosomes is a key event in many processes required for host defense. For example, CD1 antigen-presenting molecules bind microbial lipids in lysosomes and in specialized compartments formed from the fusion of phagosomes with lysosomes to form phagolysosomes (Hava et al., 2008; Ramachandra et al., 2009). After synthesis in the endoplasmic reticulum (ER) and delivery to the cell surface, CD1 molecules are internalized into the endocytic system where they bind lipid antigens and then carry them back to the cell surface to stimulate T cell activation (Cohen et al., 2009). Previous studies on CD1 trafficking defined tyrosine-based sorting motifs in the tails of CD1 isoforms (CD1b, CD1c, and CD1d), which bind adaptor protein 2 (AP-2) and mediate their internalization into the early endocytic system via clathrin-coated pits. The tails of CD1b and mCD1d bind adaptor protein 3 (AP-3), which sorts them into late endosomes and lysosomes (Cernadas et al., 2003; Chiu et al., 2002; Elewaut et al., 2003). Entry into lysosomes is critical for access to saposins, which load lipids into CD1 molecules, and for access to degradative enzymes that processes microbial lipid antigens (Cohen et al., 2009).

Understanding how trafficking of antigen-presenting molecules is directed to lysosomes and phagolysosomes is critical to understanding host defense. A number of molecules that drive the steps involved in vesicular trafficking have been described, but few have been implicated in regulating lysosomal traffic in mammalian cells. In yeast, molecules of the vacuole protein sorting (VPS) class have been described to play a role in trafficking to the vacuole, an organelle analogous to the lysosome. However, the role of many VPS proteins in mammalian lysosomes remains unknown. Small GTPases of the Ras-superfamily such as Rabs and ADP ribosylation factors (Arfs) serve as the vesicle “signposts” and organizers of membrane traffic and help mediate vesicle budding and recruitment of effector proteins (Behnia and Munro, 2005). For example, Rab7 has been proposed to control trafficking from late endosomes to lysosomes through recruitment of effectors that control a dynein-dynactin motoring apparatus (Zhang et al., 2009).

To identify molecular mediators of lysosomal trafficking, we developed a shRNA library targeting trafficking molecules and used it in a screen for loss of lysosome-dependent CD1 antigen-presenting function. Our screen identified Arl8b as a strong mediator of CD1 trafficking to lysosomes and antigen presentation.

Arl8b is a small GTPase of the Arl (*Ar*f-*l*ike) family. Previous reports implicate Arl8b in the distribution of lysosomes within the cytosol (Bagshaw et al., 2006; Hofmann and Munro, 2006). Here, we identified a role for Arl8b directing endosome to lysosome trafficking for multiple types of cargo. Arl8b silencing resulted in a delay in delivery of fluid phase dextran, receptor-internalized low-density lipoprotein (LDL), and CD1 cargo to lysosomes. Furthermore, Arl8b was found to bind and recruit the VPS41 subunit of the HOPS (*ho*motypic fusion and *p*rotein *s*orting) complex to lysosomes. Arl8b silencing and loss of HOPS complex subunit recruitment to lysosomes resulted in a defect in trafficking of CD1 molecules to lysosomes and delayed formation of CD1-lipid antigen complexes. Importantly, phagosome to lysosome delivery and fusion were delayed by Arl8b silencing resulting in a defect in microbial killing. Together, these data mark Arl8b as a regulator of endosome to lysosome trafficking pathways of special significance for host defense.

## Results

### CD1d Lysosome-Dependent Antigen Presentation Screen Identifies Arl8b

**Figure 1:**
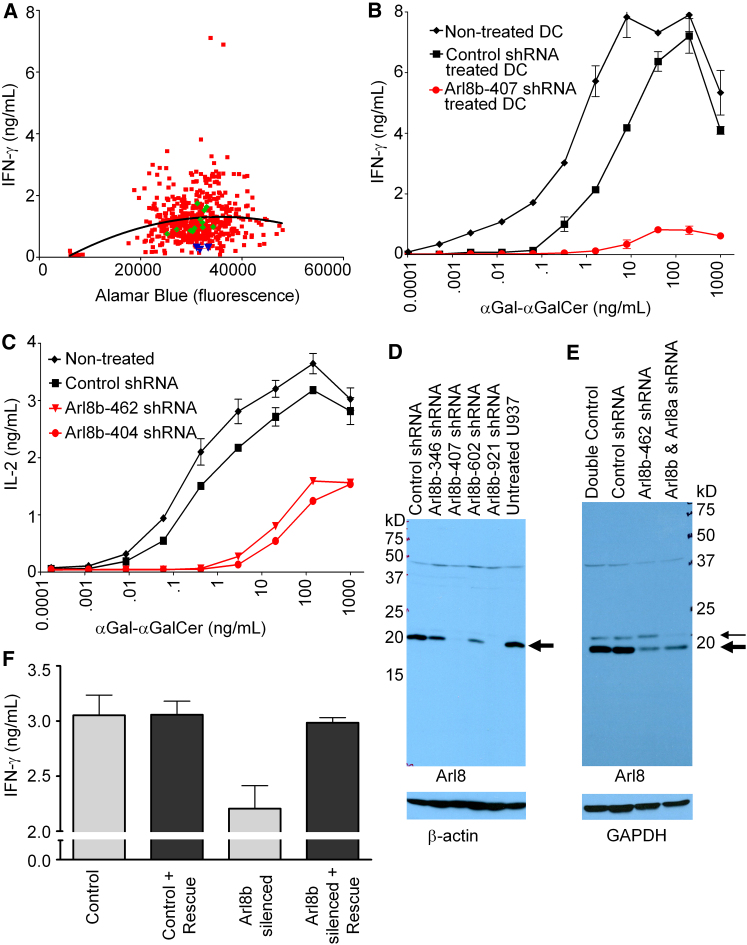
shRNA Silencing of Arl8b Decreases CD1d Antigen Presentation (A) Composite data from two sample screening plates. The number of U937 cells surviving drug selection (Alamar Blue) was plotted versus the response of NKT cells (IFN-γ). A best fit line was drawn for each batch of screening plates and shRNA constructs compared to the line to measure the effect on CD1d presentation. Experimental shRNAs (red squares), shRNAs targeting Arl8b (blue triangles), and control shRNAs included on each screening plate targeting GFP, RFP, and LacZ (green diamonds) are shown. (B) Arl8b silenced and control DCs were plated with αGal-αGalCer in the indicated doses along with NKT-cell clone BM2a.3. Following overnight incubation NKT-cell stimulation was assessed by IFN-γ ELISA. (C) Arl8b silenced and control murine RAW macrophages were plated with αGal-αGalCer along with NKT-cell hyrbidoma DN32. Following overnight incubation DN32 stimulation was assessed by IL-2 ELISA. (D and E) Immunoblots of U937 (D) or RAW (E) lysates made in 0.5% Triton blotted with Arl8b antisera (upper panel) or stripped and reprobed for loading controls (lower panel). The arrow indicates the position of the dominant band inferred to be Arl8b, with a M_r_ of 19.5 kDa based on standards. The lighter arrow indicates the inferred position of Arl8a. (F) HeLa CD1d-expressing cells were stably transduced with control or shArl8b-407 targeting shRNA and subsequently transfected with the construct used in F-containing silent mutations in the shRNA target sequence, termed “rescue.” αGal-αGalCer lipid was then added at 1 ng/mL along with BM2a.3 the response measured by IFN-γ ELISA. All error bars indicate standard error of the mean. See also Figure S1.

We built a custom shRNA trafficking library to screen for lysosomal dependent antigen presentation by CD1d. CD1d^+^ U937 myelomonocytic cells were transduced with shRNA and stably selected with puromycin, then incubated with α(1→2)GalαGalCer (αGal-αGalCer), a model lipid antigen that requires lysosomal cleavage of the terminal αGal to reveal the antigenic epitope αGalCer (Prigozy et al., 2001). We accessed formation and trafficking of CD1d⋅αGalCer complexes by measuring IFN-γ secretion from CD1d-restricted αGalCer reactive natural killer T cells (NKT-cells). By plotting the IFN-γ response of NKT-cells against the number of surviving U937 cells, we can readily identify wells in which shRNA silencing of a trafficking molecule negatively impacts NKT-cell activation by reduced NKT-cell secretion of IFN-γ (Figure 1A). For validation of the shRNA screen, the library included several control shRNA expected to inhibit αGalCer presentation. Strong reduction in NKT cell activation was noted for shRNA targeting adaptor protein-2 (AP-2) and clathrin heavy chain, both of which control CD1d internalization and for shRNA targeting prosaposin that yields the lysosomal saposins necessary to transfer lipids to CD1 (Figure S1 available online).

Importantly, a number of library shRNAs either blocked or enhanced antigen presentation to NKT cells. Two unique shRNA sequences targeting Arl8b (Arl8b-346 and Arl8b-407) that markedly reduced the ability of U937 cells to stimulate IFN-γ secretion from NKT Cells were selected from the screen for further study (Figure 1A, blue triangles). Given that library screening was performed with the U937 tumor cell line, we extended this finding by using primary human monocyte-derived dendritic cells (DCs) as professional antigen-presenting cells (APCs). APCs were stably transduced with lentivirus expressing Arl8b-407 shRNA or control shRNA, plated with increasing doses of αGal-αGalCer lipid antigen, and the response of primary human NKT-cells (clone BM2a.3) was measured by IFN-γ secretion. Although Arl8b silencing did not change CD1d at the cell surface, it produced a two-log decrease in dose-dependent antigen presentation compared to control shRNA treated DCs (Figure 1B, e.g., the stimulation achieved by .32 ng/mL αGal-αGalCer in control DCs is achieved only by adding 40 ng/mL αGal-αGalCer in Arl8b-silenced DCs). The maximal stimulation reached at the highest antigen dose in Arl8b silenced cells was only 11% of control cells. By comparison, Arl8b silencing did not alter cell surface expression of CD1a and showed normal stimulation of CD1a-autoreactive clones, suggesting Arl8b silencing did not impact CD1a endocytic pathways (data not shown). Note that there was a slight decrease in CD1d presentation due to lentiviral transduction alone, consistent with the results of others that viral infection itself can modestly impact CD1d presentation (Chen et al., 2006; Yuan et al., 2006). Subsequent experiments verified that Arl8b silencing reduced CD1d presentation of αGal-αGalCer in multiple cell types including DCs, B lymphoblastoid cells (C1R), CD1 transfected cells (HeLa), and myelomonocytic cells (U937), suggesting that the Arl8b pathway is likely to be common in many and possibly all cell types.

To extend the evaluation to a second species, we examined murine CD1d (mCD1d), which predominantly traffics through lysosomal compartments. We identified shRNAs targeting murine Arl8b that were distinct from those against human Arl8b. We next transduced RAW macrophages with lentivirus encoding these shRNAs and measured dose response curves to αGal-αGalCer. Consistent with findings for human CD1d, silencing of murine Arl8b led to more than a two-log decrease in dose-dependent mCD1d presentation in RAW macrophages and 39% maximal stimulation of NKT-Cell hybridomas at the highest concentrations of antigen dose tested (Figure 1C).

To verify that the shRNA sequences reduced Arl8b mRNA expression, we performed reverse transcriptase-polymerase chain reaction (RT-PCR) with Arl8b-specific primers. Arl8b targeting shRNA reduced expression of the target by 74% (Arl8b-346) and 85% (Arl8b-407) for human Arl8b and 85% (Arl8b-461) and 90% (Arl8b-404) for murine Arl8b, respectively, although they did not lead to changes in mRNA amounts of other Arls tested (e.g., Arl8a, Arl2, and Arl13b). Further, we raised a rabbit antiserum against the C-terminal peptide of Arl8b to assess Arl8b protein amounts by western blotting. Because human and murine Arl8b are 100% identical in amino acid sequence, the same antiserum was used for detecting Arl proteins of both species. The antiserum recognized a single dominant band in human U937 cell lysates that was reduced by >95% upon Arl8b silencing. The antiserum recognized a doublet in RAW macrophages of which the dominant lower band, equivalent in migration to the band observed in U937, was also specifically reduced (Figures 1D and 1E). Overexpression of Arl8b cDNA led to a dramatic increase in a band of the same mobility confirming its identity as Arl8b (data not shown).

Even when silencing the intended target, RNA interference approaches can give false-positive results through off-target effects. To address this caveat, we generated a panel of unique shRNAs targeting Arl8b along the length of the transcript, with the reasoning that multiple unique sequences were unlikely to have similar off target effects. A panel of six distinct shRNA hairpins all produced reductions in Arl8b mRNA and CD1d presentation by U937 cells (Figure S1). In a second approach, HeLa cells were transduced with Arl8b shRNA and subsequently transfected with Arl8b cDNA containing silent mutations generating a 6/21 mismatch to the Arl8b shRNA. We found that reintroduction of Arl8b via cDNA expression corrected the defect in HeLa CD1d presentation, further confirming that the observed phenotypes were due to an on-target effect specific to Arl8b silencing (Figure 1F).

### Arl8b Silencing Induces a Defect in Cargo Delivery to Lysosomes

**Figure 2:**
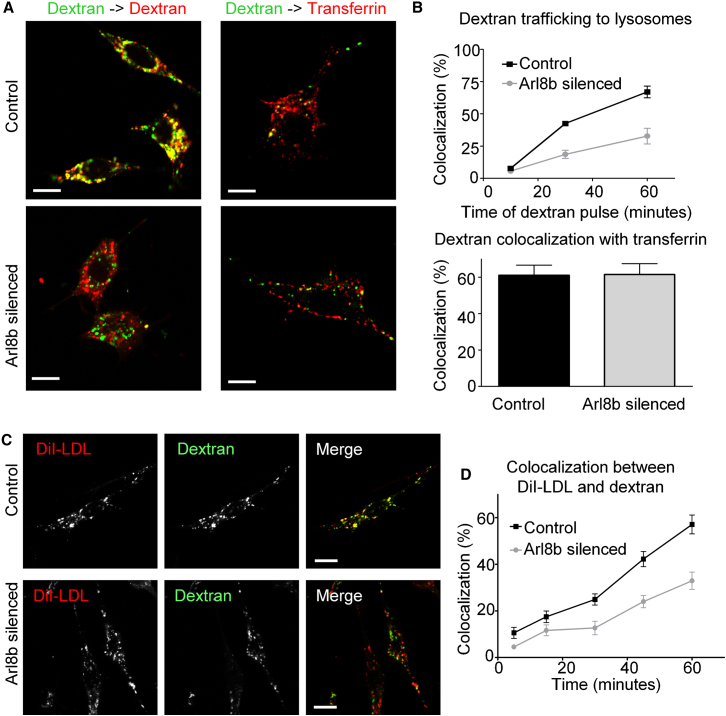
Arl8b Silencing Results in Delayed Delivery of Dextran and LDL to Lysosomal Compartments (A) Lysosomes of Arl8b-silenced and control RAW cells were labeled with dextran (Alexa Fluor 546, red) pulsed for 60 min and then underwent a >6 hr chase to allow accumulation in lysosomes (left). Cells were then incubated with a second dextran (Alexa Fluor 488, green) for various times (10 min, 30 min, 60 min) and colocalization of both dextrans was assessed (right). Alternatively, cells were prelabeled for 30 min with transferrin (Alexa Fluor 546) to mark early endocytic compartments and were subsequently pulsed with dextran (Alexa Fluor 488) in the continued presence of transferrin for short time points (5 min, 10 min, 30 min). Shown are representative images for 30 min of dextran → dextran pulse (left panels) and 10 min of dextran → transferrin pulse (right panels). (B) Colocalization quantification for >20 cells for each condition at each time point are shown for dextran → dextran pulse (top panel) at 10 min, 30 min, and 60 min and dextran → transferrin pulse (bottom panel) measured at 10 min. (C) Lysosomes of Arl8b silenced and control cells were prelabeled with dextran (Alexa Fluor 488, 1 hr pulse followed by 6 hr chase). Cells were subsequently starved in serum-free medium for 2 hr and then incubated with DiI-LDL (red) for up to 30 min. The 60 min time point represents 30 min of pulse followed by 30 min of chase in serum-free medium. Representative images from 60 min are shown. (D) Colocalization quantification of DiI-LDL with dextran in Arl8b-silenced and control cells. All error bars indicate standard error of the mean. See also Figure S2.

Arl GTPases localize to specific subcellular compartments and help to define organelle identity and direct organelle traffic. Staining with Arl8b antiserum localized endogenous Arl8b to lysosomal associated membrane protein-1 (LAMP1)^+^ lysosomes (Figures S2A and S2B). Given the profound decrease in CD1d antigen presentation observed in Arl8b silenced cells (Figures 1B and 1C) and the importance of trafficking to the lysosome as a key step in antigen presentation, we hypothesized Arl8b might control traffic into lysosomes. To address this possibility we utilized fluorophore conjugates of dextran, a carbohydrate molecule taken up into cells by fluid phase pinocytosis. Arl8b-silenced and control cells were pulsed with an Alexa Fluor 546 (red) conjugate of dextran followed by a long chase (>6 hr) to allow dextran to traffic to and accumulate in lysosomes. Cells were then pulsed with a dextran molecule conjugated to a second color (Alexa Fluor 488 green) and the amount of colocalization with the first dextran (red) assessed over time via confocal microscopy (Figure 2A, left panels). Arl8b-silenced cells showed a 56% reduced colocalization of the two dextrans when compared with control cells at 30 min following pulse of Alexa Fluor 488 dextran and 51% reduced colocalization at 60 min following the pulse, indicating a delay in fluid phase cargo trafficking into lysosomes (Figure 2B, top panel).

To extend these findings to cargo taken up by receptor mediated endocytosis, we assessed the delivery of DiI-labeled LDL (3,3′-dioctadecylindocarbocyanine-low density lipoprotein) molecules to lysosomes. After uptake by the LDL-R (LDL receptor), LDL disassociates from LDL-R. Whereas LDL-R recycles back to the cell surface, LDL continues trafficking to lysosomes. Lipid antigens such as α(1− > 2)GalαGalCer are also taken up into cells through binding LDL-R and probably follow a LDL-directed trafficking route to lysosomes (van den Elzen et al., 2005). To assess this pathway, we prelabeled lysosomes of Arl8b silenced and control cells with a pulse of Alexa Fluor 488 (green) dextran and then with a chase (chase time > 4 hr). We subsequently serum starved cells for >2 hr to allow LDL-R to accumulate on the cell surface and to synchronize LDL uptake. A DiI-LDL pulse in serum-free media was given to cells followed by a chase in DiI-LDL-free media for various times and the colocalization of DiI-LDL with lysosomal dextran was assessed (Figures 2C and 2D). At 30 min after the DiI-LDL pulse, colocalization was reduced by 52% in Arl8b-silenced cells compared to control cells, indicating a delay in arrival of LDL to lysosomal compartments. A similar delay was noted for the arrival of antibody-bound CD1d molecules to lysosomes in Arl8b-silenced cells (Figure S2C). Together, these experiments suggest that Arl8b plays an important role in delivery of multiple types of cargo to lysosomes.

In contrast to the effects of Arl8b on trafficking to lysosomes, trafficking through early recycling endosomes assessed by the transient colocalization of dextran with the early endocytic marker transferrin receptor (TfR) and total transferrin uptake by TfR were unaffected in Arl8b-silenced cells (Figure 2A, right panels and Figure 2B, bottom panel). Thus, the trafficking defect of cargo to lysosomes in Arl8b-silenced cells occurred downstream of transferrin receptor-positive early endocytic compartments. In contrast to a delay in trafficking, Arl8b-silenced cells did not display changes in lysosomal acidification compared to control cells, nor did they display differences in the steady-state localization of several markers in lysosomes (saposins, cathepsin D, Rab9, Figure S2D).

Additionally, we confirmed previous reports implicating Arl8b in the distribution of lysosomes within the cell (Figure S3A). The distribution of lysosomes correlated with combined expression of both Arl8a and Arl8b (Figure S3B). However, Arl8 double-silenced cells appeared to be morphologically unhealthy in light microscopy so we continued to focus studies on Arl8b-silenced cells rather than Arl8 double-silenced cells.

### Arl8b Silencing Does Not Alter the Recruitment of Rab7 or Its Effectors to Lysosomes

**Figure 3:**
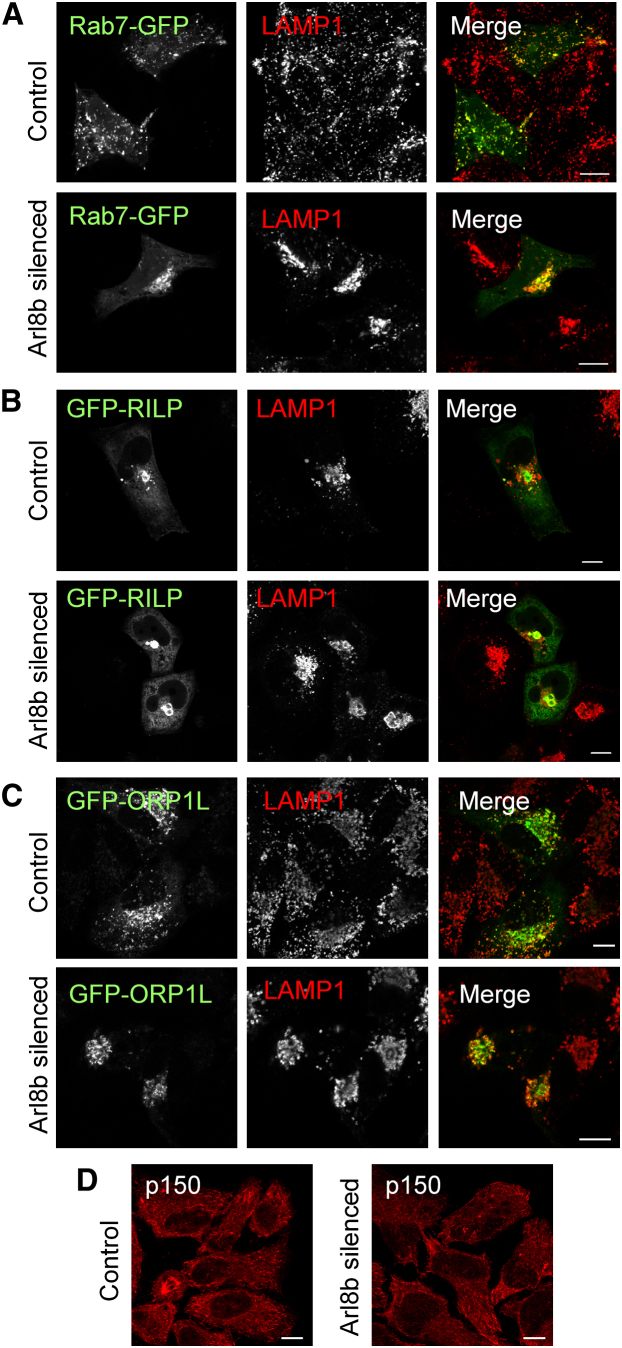
Arl8b Silencing Does Not Alter the Recruitment of Rab7 or Its Effectors to Lysosomes Control or Arl8b-silenced HeLa cells were transfected with the indicated constructs (left panels), plated on glass coverslips, fixed, permeabilized, and stained for LAMP1 (middle panels). Merged images are shown (right panels). (A) Colocalization between Rab7-GFP and LAMP1 in control cells (top row) and Arl8b-silenced cells (bottom row). (B) Colocalization between RILP-GFP and LAMP1 in control cells (top row) and Arl8b-silenced cells (bottom row). (C) Colocalization between GFP-ORLP1 and LAMP1 in control cells (top row) and Arl8b-silenced cells (bottom row). (D) Distribution of the dynactin motor subunit p150 in control cells (left) and Arl8b-silenced cells (right).

The small GTPase Rab7 directs trafficking from late endosomes into lysosomes through the recruitment of the effector RILP (*R*ab7 *i*nteracting *l*ysosomal *p*rotein) (Cantalupo et al., 2001). In turn, the Rab7-RILP complex binds p150, a subunit of a dynein-dynactin motoring apparatus (Johansson et al., 2007). This step is regulated in part by the oxysterol binding protein ORP1L (Rocha et al., 2009). The Rab7⋅RILP⋅ORP1L⋅p150 complex drives movement toward the minus end of microtubules. Therefore, changes in expression of Rab7 or its effectors functionally impacts trafficking into lysosomes through control of lysosomal localization. Given our identification of a role for Arl8b in directing both traffic into lysosomes (Figure 2) and the localization of lysosomes (Figure S3), we designed experiments to determine if Arl8b might function through the recruitment of Rab7 or its effectors to lysosomes. Arl8b-silenced and control cells were transfected with Rab7-green fluorescent protein (GFP), plated on glass coverslips, fixed, and analyzed for the distribution of LAMP1 and Rab7. In control cells, Rab7 localized almost exclusively to LAMP1^+^ vesicles (Figure 3A, top row). Similarly, in Arl8b-silenced cells Rab7 continued to colocalize strongly with LAMP1 even though lysosomes were redistributed, indicating that Arl8b is not required for localization of Rab7 to lysosomes (compare right panels, Figure 3A). Rab7 recruits a subset of RILP molecules to late endosomes. Both control and Arl8b-silenced cells displayed similar amounts of RILP recruitment to lysosomes as measured by colocalization of GFP-RILP with LAMP1 (compare right panels, Figure 3B). Likewise, recruitment of ORP1L molecules to LAMP1^+^ lysosomes appeared unchanged in Arl8b-silenced cells when compared to control cells (Figure 3C). Arl8b silencing also did not alter the distribution of p150, the dynein-dynactin motor bound by Rab7-RILP (Figure 3D). Together, these data suggest that Arl8b does not direct lysosomal trafficking by controlling the localization of Rab7 to lysosomes or its function in recruiting effectors to this compartment. Rather, Arl8b is likely to function through other effectors.

### Arl8b Recruits the HOPS Complex Member VPS41 to Lysosomes

**Figure 4:**
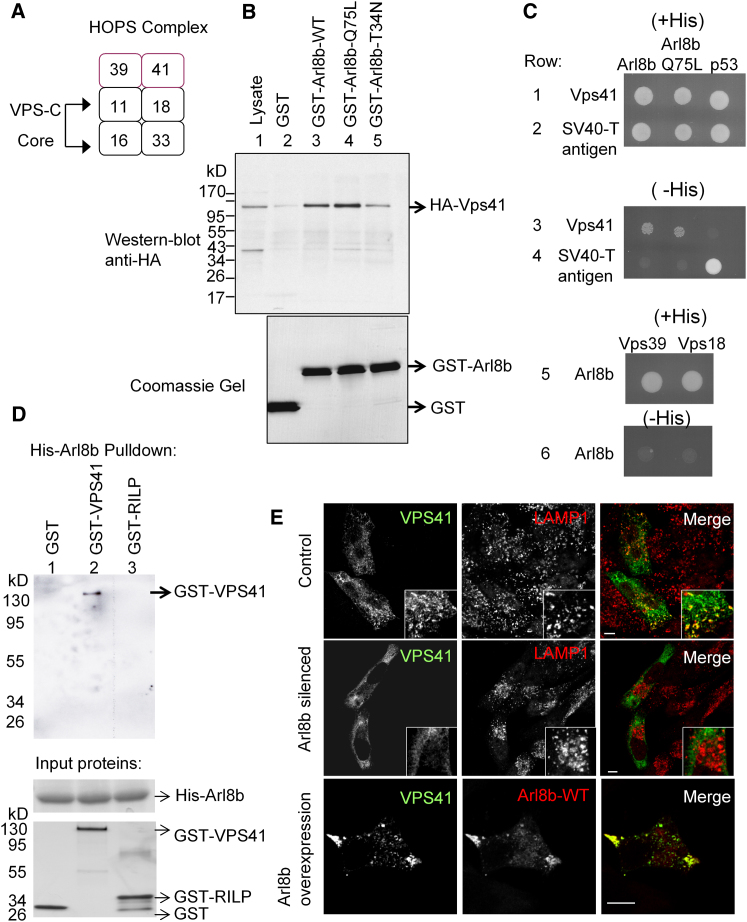
Arl8b Recruits the HOPS Complex Member VPS41 to Lysosomes (A) Adapted from Nickerson et al. (2009). Proposed subunits of the mammalian HOPS complex based on orthology to *S. cerevisiae*. The VPS-C core is composed of hVPS11, hVPS18, hVPS16, and hVPS33. In yeast, VPS39 and VPS41 combine with VPS-C to form HOPS. The HOPS complex regulates trafficking to lysosomes in *S. cerevisiae*. (B) Lysates from HeLa cells transfected with HA-VPS41 were run over glutathione-conjugated beads bound to GST (lane 2), GST-Arl8b (WT, lane 3), GST-Arl8b-Q75L (dominant-active mutant, lane 4), GST-Arl8b-T34N (dominant negative mutant, lane 5) and immunoblotted anti-HA. Equal amounts of lysate were run on a separate SDS-PAGE gel stained with Coomassie Brilliant Blue for total protein detection. (C) Yeast two-hybrid analysis. Arl8b, dominant-active Arl8b Q75L, and p53 were cloned into the DNA-binding domain vector (Matchmaker, Clonetech). VPS41, SV40-T, VPS39, and VPS18 were cloned into the activation domain vector. Yeast were plated on nonselective medium (+His) to confirm viability and plated on selective medium (−His) to detect interactions. (D) Interaction of Arl8b with VPS41 in two-protein system. Purified Histadine-tagged Arl8b (shown by Coomassie stain, middle) was immobilized on a cobalt-resin column and exposed to purified GST (lane 1), GST-VPS41 (lane 2), or GST-RILP (lane 3) (shown by silver stain, bottom). Eluates were run on SDS-PAGE and blotted anti-GST. (E) Control (top row), Arl8b-silenced (middle row), and Arl8b-overexpressing (bottom row) HeLa cells were transfected with HA-VPS41 and analyzed in confocal microscopy for the distribution of VPS41 to LAMP1^+^ compartments. For a list of GST-Arl8b interacting proteins identified, see also Figure S4.

To discover effectors of Arl8b, we conducted biochemical pull-downs with GST-Arl8b (glutathione- S-transferase-tagged Arl8b) and identified candidate Arl8b effectors through mass spectrometry. A list of Arl8b interacting proteins is given in Figure S4. Strikingly, we identified peptides corresponding to mammalian orthologs of the *Saccharomyces cerevisiae* HOPS complex. In yeast, the HOPS complex is composed of six subunits that together regulate all trafficking into the yeast vacuole (Figure 4A) (Nickerson et al., 2009). Four of these subunits, VPS11, VPS16, VPS18, and VPS33, constitute a core complex termed VPS-C. In mammalian cells, both VPS-C and VPS39 may be shared with early endocytic trafficking complexes and may not be specific for trafficking into lysosomes (Nickerson et al., 2009). Thus, we initially determined whether Arl8b directed trafficking to lysosomes by binding and recruiting mammalian VPS41. Cells were transfected with HA-VPS41 (hemagglutinin-tagged VPS41) and lysates were passed over glutathione beads bound to either GST alone or GST-Arl8b. Eluates were resolved on SDS-PAGE, immunoblotted for HA-VPS41, and Coomassie stained (Figure 4B, lanes 1–3). GST-Arl8b bound VPS41 whereas GST did not (compare strength of band in lane 3 to those in lanes 1 and 2). Because Arl8b is a GTPase, it cycles between active GTP-bound and inactive GDP-bound forms. Previously, both a dominant-active GTP-locked form of Arl8b (Arl8b-Q75L) and a dominant-negative GDP-locked form of Arl8b (Arl8b-T34N) have been described (Hofmann and Munro, 2006; Okai et al., 2004). Pull-down with GST-Arl8b-Q75L confirmed that dominant-active Arl8b promoted interaction with VPS41 (Figure 4B, note increase in intensity of VPS41 band in lane 4 compared to lane 3). Conversely, GST-Arl8b-T34N negated the interaction with VPS41 compared to GST-Arl8b-WT and GST-Arl8b-Q75L (Figure 4B, compare loss of strength of VPS41 band in lane 5 to those in lanes 4 and 3). These data argue that Arl8b binds VPS41 in manner dependent on its GTP versus GDP bound state, suggesting VPS41 is an effector of Arl8b.

To determine whether Arl8b bound VPS41 directly, we undertook two approaches. In the first, both Arl8b and HOPS complex members were cloned into DNA binding and transcription activation domain vectors, respectively, in standard yeast two-hybrid complementation systems. All strains were viable as evidenced by their growth on nonselective media (Figure 4C, rows 1, 2, and 5 “+His”). Both WT Arl8b and dominant -active Arl8b (Q75L) bound VPS41, as evidenced by their growth on selective media (Figure 4C, row 3 “–His”). In contrast, Arl8b did not bind VPS39 or VPS18 as evidenced by the failure of growth in selective media (Figure 4C, row 6 “–His”). As expected, p53 bound the positive control SV40-T but did not bind VPS41 (Figure 4C, rows 3 and 4). Together, these data argue that Arl8b specifically binds VPS41. To confirm this finding, we expressed and purified Histadine-tagged Arl8b (His-Arl8b), GST-tagged VPS41, and GST bound to the Rab7 binding domain of RILP (GST-RILP). His-Arl8b was immobilized on cobalt resin and incubated with GST, GST-VPS41, and GST-RILP. Eluates were run on SDS-PAGE and immunoblotted for GST. We found Arl8b-bound VPS41 (lane 2) but did not bind either GST (lane 1) alone or GST-RILP (lane 3) (Figure 4D, upper blot). These experiments demonstrate that Arl8b directly binds VPS41.

To determine whether Arl8b recruits VPS41 to lysosomes, we examined the distribution of VPS41 in control, Arl8b-silenced, and Arl8b-overexpressing cells (Figure 4E). In control cells, VPS41 showed partial colocalization with LAMP1, indicating that a subset of VPS41 molecules was present on lysosomes (Figure 4E, first row, right panel). However, in Arl8b-silenced cells no colocalization between VPS41 and LAMP1 was detected (Figure 4E, second row, right panel). This suggests that VPS41 is not recruited to lysosomes in the absence of Arl8b. Conversely, overexpression of Arl8b led to a dramatic recruitment of VPS41 to Arl8b^+^ LAMP1^+^ lysosomes (Figure 4E, third row). Together, these data argue that Arl8b controls the localization and recruitment of the HOPS complex member VPS41 to lysosomes and that VPS41 is a direct effector of Arl8b.

### Arl8b and VPS41 Recruit Other HOPS Complex Members to Lysosomes

**Figure 5:**
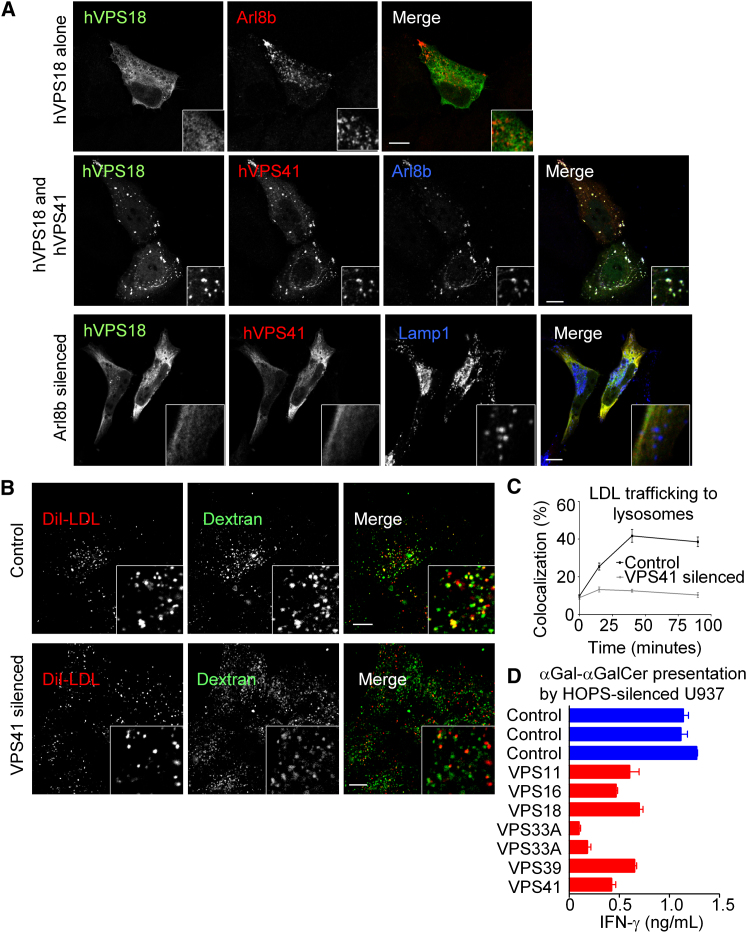
Other Components of the HOPS Complex Are Recruited to Lysosomes by Arl8b and hVPS41 (A) Control HeLa cells (top row) were transfected with hVPS18 and Arl8b, fixed, and stained for hVPS18 (left) and Arl8b (middle). In the middle row, cells were transfected with hVPS41 (middle, Arl8b staining at right) in addition to hVPS18. In the bottom row, Arl8b-silenced HeLa cells were transfected with hVPS18 (left), hVPS41 (middle) and Arl8b (right), fixed, and stained. Similar data were obtained with VPS11 and VPS16 (Figure S5). (B) Similar to Figure 2C, lysosomes of hVPS41 silenced cells were prelabeled with dextran conjugated to Alexa Fluor 488 (green). Cells were then starved to allow LDL-R to accumulate, pulsed with DiI-LDL (red) for 15 min, and chased in DiI-LDL free medium for varying times, and the trafficking of LDL to lysosomes was assessed by colocalization with dextran. Shown are representative images of LDL trafficking to lysosomes after 90 min of chase time in control cells (top row) and hVPS41-silenced cells (bottom row). (C) Quantification of the colocalization between dextran (green) and LDL in control and hVPS41-silenced cells was performed for >30 cells at each indicated time point. (D) Indicated HOPS complex members were stably silenced by shRNA treatment in CD1d^+^ U937 cells as described (see Experimental Procedures). Cells were then pulsed with 70 ng/mL αGal-αGalCer, 50,000 NKT cells (J3N.5) added, and the combination was incubated overnight. Stimulation of NKT-cells was assessed by IFN-γ cytokine Elisa. A list of targeting shRNA sequences is given in the Supplemental Information. All error bars indicate standard error of the mean. See also Figure S5.

VPS-C subunits of the HOPS complex did not show appreciable localization to LAMP1^+^ lysosomes when HA-tagged versions were expressed in cells (Figure 5A, Figure S5, and data not shown). Instead, they primarily localized to the cytosol with partial distribution to LAMP1 negative punctae. Overexpression of Arl8b alone led to only slight changes in the distribution of VPS-C molecules. However, when both Arl8b and hVPS41 were expressed in cells, VPS18 showed a dramatic recruitment to lysosomes (Figure 5A, middle row), suggesting that Arl8b acts together with VPS41 to promote the recruitment of VPS18. Further, in Arl8b-silenced cells the localization of both VPS41 and VPS18 to lysosomes was lost, suggesting that recruitment of HOPS complex members to lysosomes depends on Arl8b (Figure 5A, bottom row). Similar data were obtained for VPS11 and VPS16 (Figure S5). Together, these data support a model in which Arl8b primarily recruits VPS41, and together Arl8b and VPS41 promote recruitment of the other HOPS complex members to lysosomes.

### The HOPS Complex Directs Trafficking into the Mammalian Lysosome

Because HOPS complex members are known to direct trafficking to the yeast vacuole, we assessed whether loss of HOPS impacted trafficking to the mammalian lysosome. As in Figures 2C and 2D, lysosomes of VPS41-silenced or control cells were prelabeled with dextran and pulsed with LDL. After chase, arrival of LDL in lysosomes was assessed through colocalization with dextran. Similar to results for Arl8b-silenced cells, we found VPS41-silenced cells displayed a dramatic delay in delivery of LDL to lysosomes when compared to controls (Figure 5B, images, Figure 5C, quantification).

To assess whether loss of the HOPS complex recruited by Arl8b resulted in a functional defect in CD1d presentation, we took advantage of our trafficking shRNA library that contained sequences directed against HOPS complex members. Multiple unique shRNA sequences targeting HOPS gave reductions in CD1d antigen presentation compared to control sequences (Figure 5D). In particular, shRNA sequences targeting VPS33A gave strong reductions in CD1d presentation of αGal-αGalCer and were among the strongest hits in the trafficking library. Together, these data suggest that Arl8b recruits the HOPS complex to lysosomes, which in turn directs cargo trafficking into this compartment with consequences for CD1d antigen presentation.

### Arl8b Silencing Leads to a Delay in Lipid Antigen Binding to CD1d and Delivery of Antigen-Bound Complexes to the Cell Surface

**Figure 6:**
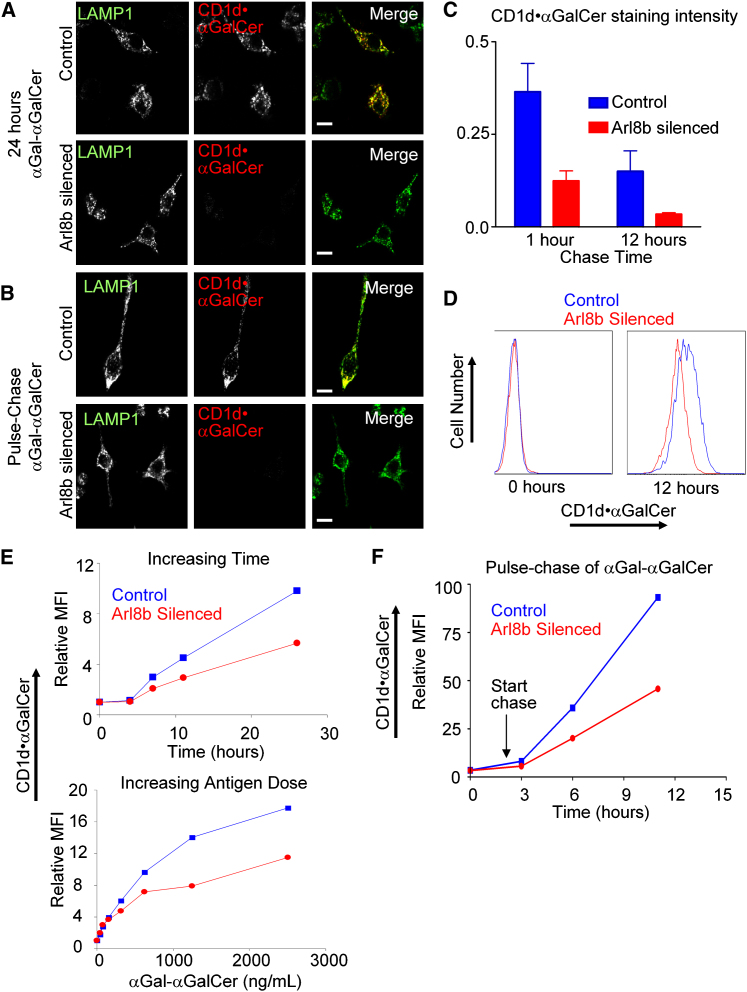
Arl8b-Silenced Cells Show Delayed Formation of CD1d⋅αGalCer Complexes (A) Arl8b-silenced and control RAW cells were cocultured with 500 ng/mL αGal-αGalCer for 24 hr, costained with L363 (CD1d⋅αGalCer complex specific) and LAMP1 mAbs, and analyzed by confocal microscopy. (B) Cells were pulsed with 2.5 μg/mL αGal-αGalCer for 2 hr and then underwent a washout and replacement with fresh medium (“chase”). Representative images are shown for 1 hr of chase. (C) Quantification of CD1d⋅αGalCer staining for >30 Arl8b-silenced and control cells at the indicated time points. The average pixel intensity for CD1d⋅αGalCer staining per cell was divided by the average LAMP1 pixel intensity for the same cell to normalize. Error bars indicate the standard error of the mean. (D) Arl8b-silenced and control cells were cocultured with 500 ng/mL αGal-αGalCer for 0 (left panel) or 12 (right panel) hr and CD1d⋅αGalCer complexes at the cell surface were analyzed by flow cytometric staining with mAb L363. (E) Arl8b-silenced and control cells were cocultured either with 500 ng/mL αGal-αGalCer for increasing amounts of time (upper panel) or for 12 hr with increasing concentrations of αGal-αGalCer (lower panel), and CD1d⋅αGalCer complexes at the cell surface were analyzed. Mean fluorescence intensities (MFIs) relative to time 0 were plotted for staining with L363 mAb. (F) Instead of continuous coculture, Arl8b-silenced and control cells were pulsed with 2.5 μg/mL αGal-αGalCer for 2 hr and then chased in complete media for 1 hr, 4 hr, and 9 hr. The appearance of CD1d⋅αGalCer complexes at the cell surface was monitored by L363 flow cytometric staining and MFIs plotted.

A delay in trafficking into lysosomes in Arl8b- or HOPS-silenced cells might lead to a delay in the formation of CD1d⋅αGalCer complexes for immune recognition by NKT-cells. To examine the formation of CD1d⋅αGalCer complexes directly, we utilized a monoclonal antibody (L363) that specifically recognizes the CD1d⋅αGalCer complex but not CD1d without this bound antigen or CD1d bound to unprocessed αGal-αGalCer (Yu et al., 2007). Cells were cocultured with 500 ng/mL αGal-αGalCer and examined in confocal microscopy with mAb L363 and anti-LAMP1 for assessment of the formation of CD1d⋅αGalCer complexes intracellularly. Eight hours after αGal-αGalCer coculture, CD1d⋅αGalCer complexes could be detected in lysosomes of control cells, whereas they were largely absent in Arl8b silenced cells (data not shown). This difference was even more pronounced 24 hr after coculture with αGal-αGalCer, indicating that the defect in cargo delivery to lysosomes was mirrored by a defect in CD1d⋅αGalCer complex formation in lysosomes (Figure 6A). Cells were also examined after a short pulse of αGal-αGalCer at higher concentration (2.5 μg/mL for 2 hr) followed by a chase. Arl8b-silenced cells showed only 34% staining intensity of control cells at 1 hr and 21% staining intensity of control cells at 12 hr after αGal-αGalCer pulse-chase (Figures 6B and 6C), confirming the delay in formation of CD1d⋅αGalCer complexes.

The delay in formation of CD1d⋅αGalCer complexes intracellularly in Arl8b-silenced cells could lead to a corresponding delay in their delivery to the cell surface and a reduction in CD1d⋅αGalCer-restricted NKT cell stimulation, explaining the defect in CD1d antigen presentation observed in Arl8b- or HOPS-silenced cells. To assess this possibility, we cocultured cells either with αGal-αGalCer for various times or with differing amounts of αGal-αGalCer for a fixed time and assessed the appearance of CD1d⋅αGalCer complexes on the cell surface by flow cytometry with mAb L363 (Figure 6D). After 26 hr of coculture with αGal-αGalCer, Arl8b-silenced cells showed only 57% of the cell surface expression of CD1d⋅αGalCer complexes as compared to control cells (Figure 6E, upper panel). Increasing the amount of αGal-αGalCer administered did not overcome this defect (Figure 6E, lower panel, e.g., Arl8b-silenced cells show only 56% the mean fluorescence intensity (MFI) of control cells at 1250 ng/mL αGal-αGalCer). This defect was specific to CD1d⋅αGalCer complexes because amounts of mCD1d itself on the cell surface did not change during the experiment in either control or Arl8b-silenced cells. When cells were assessed with a short pulse of high concentration antigen followed by 4 or 9 hr of chase time (6 and 11 hr total time), Arl8b-silenced cells showed ∼50% of the MFI of control cells when stained for CD1d⋅αGalCer complexes by flow cytometry, confirming the results obtained with continuous pulses of αGal-αGalCer (Figure 6F). Together, these results suggest that the delay in trafficking into lysosomes leads to a delay in the formation of CD1d⋅αGalCer complexes in lysosomes and a subsequent delay in their appearance at the cell surface.

### Arl8b Silencing Leads to a Delay in LAMP1 Appearance on Phagosomes and a Defect in Microbial Killing

**Figure 7:**
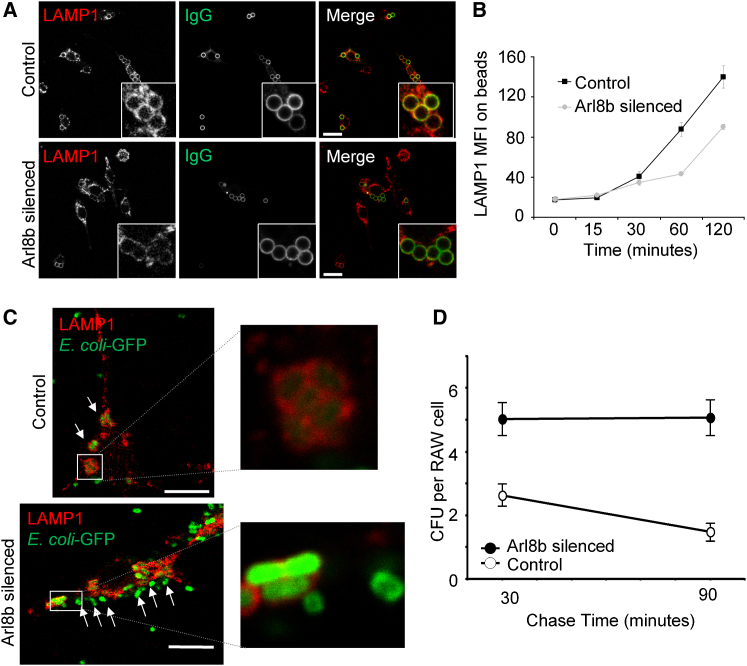
Arl8b Silencing Delays Fusion of Phagosomes with Lysosomes Leading to a Defect in Microbial Clearance (A) Control or Arl8b silenced RAW cells were plated on glass coverslips and then centrifuged with 3 μm IgG-coated latex beads for 0 min, 15 min, 30 min, 60 min, and 120 min. Cells were fixed and stained for LAMP1 (red) or IgG (green). Representative images for 60 min are shown. Higher-power magnification (inset) is shown for clarity. (B) Arl8b-silenced and control cells were plated as in (A) but without coverslips. Cells were disrupted by repeated passage through a small-gauge needle in a hypotonic lysis buffer, and latex bead-containing phagosomes recovered. These were then stained with LAMP1 antibody, analyzed via flow cytometry, and LAMP1 MFI plotted against time of bead incubation. (C) Similar to (A), only cells were infected with *E. coli* expressing a GFP plasmid at an MOI of 20. Shown are representative images taken from 1 hr after infection. On the right are high-magnification images of intracellular *E. coli* from a control cell (upper-right panel) and Arl8b-silenced cell (lower-right panel). (D) Similar to (C), however instead of fixation and analysis by microscopy, RAW cells were lysed at 30 min and 90 min of chase time in gentamycin, and live *E. coli* recovered through assaying CFU on agar plates. Error bars indicate the standard error of the mean. See also Figure S6.

In order for microbial killing to occur, phagosomes must mature into phagolysosomes through content exchange with lysosomes. Given our findings that Arl8b plays a critical role in directing the trafficking of endocytosed cargo to lysosomes, we reasoned it might also mediate the trafficking of phagocytosed cargo to lysosomes. First, the phagocytosis of microbes was modeled with latex beads coated with IgG. Arl8b-silenced and control cells were plated on glass coverslips, latex beads added, and then centrifuged to synchronize bead uptake. Cells were fixed, permeabilized, stained for LAMP1 and IgG, and analyzed by fluorescence microscopy. Over time, phagosomes containing latex beads fuse with lysosomes resulting in LAMP1^+^ “rings” forming around the beads as the phagosomes mature into phagolysosomes. A striking reduction in LAMP1^+^ staining intensity in rings surrounding beads occurred in Arl8b-silenced RAW cells compared with beads in control cells (Figure 7A).

For further verification that bead phagosomes were not fusing with lysosomes in Arl8b-silenced cells, injested bead-phagosome complexes were repurified from cells and analyzed for acquisition of lysosomal markers by flow cytometry (Hmama et al., 2004). Sixty minutes after adding beads, bead phagosomes isolated from Arl8b silenced RAW revealed 49% of the LAMP1 MFI as control cell bead phagosomes (Figure 7B). Thus, acquisition of a lysosomal marker indicative of phagosome fusion with lysosomes is delayed upon Arl8b silencing. Importantly, acquisition of transferrin receptor on bead phagosomes was not affected by Arl8b silencing, again suggesting the trafficking defect occurred downstream of the early endocytic system (Figure S6B). Treatment with the actin polymerization inhibitor cytochalasin D blocked the acquisition of LAMP1 on latex beads and eliminated differences between Arl8b-silenced and control cells, suggesting LAMP1 acquisition on beads was due to a phagocytic process requiring actin reorganization and was not nonspecifically acquired during bead purification (Figure S6A). Together, these data suggest that Arl8b silencing leads to a defect in the interaction of lysosomes with latex bead phagosomes and a subsequent delay in forming phagolysosomes.

To extend these findings to live organisms, we utilized a pathogenic *Escherichia coli* strain expressing GFP. *E. coli* GFP were opsonized through binding of mouse serum, added to Arl8b-silenced or control RAW cells plated on glass coverslips, and infection synchronized by centrifugation of the bacteria onto the cells. RAW cells were then fixed, permeabilized, and analyzed for GFP (*E. coli*) and LAMP1 (lysosomes) in fluorescence microscopy. In control cells, LAMP1 accumulated around intracellular *E. coli* (Figure 7C, upper panels). In contrast, Arl8b-silenced RAW cells showed multiple intracellular *E. coli* with no surrounding LAMP1 (Figure 7C, lower panels). Further, *E. coli* GFP appeared brighter in Arl8b-silenced cells consistent with less degradation of the microbe and GFP. To directly measure intracellular killing of the microbe, we infected Arl8b-silenced or control cells with *E. coli* GFP as above, and at various times after infection viable *E. coli* were recovered through gentle detergent cell lysis. The detergent lysis disrupted RAW cell membranes but did not negatively impact *E. coli* viability, allowing assessment of the live bacteria remaining as colony forming units (CFUs). Arl8b-silenced cells showed at least twice as many viable bacteria as control cells at 30 min and 90 min after infection, indicating a defect in microbial killing (Figure 7D).

Taken together, our results show that Arl8b directs trafficking into lysosomes through recruitment of the HOPS complex, directs the intersection of CD1d with lipid antigens in lysosomes, and plays a role in intersecting phagosomes with lysosomes to generate phagolysosomes that kill microbes.

## Discussion

The function of Arl proteins is just beginning to be understood. Arl1 localizes to the trans-Golgi, where it binds a large coiled-coil protein termed a Golgin that functions as a molecular tether for incoming vesicles (Burguete et al., 2008; Munro, 2005). Thus, Arl1 is thought to mediate vesicle traffic entering the trans-Golgi. The data presented here suggest that Arl8b may direct analogous interactions for lysosomes through recruiting the HOPS complex. In yeast, the HOPS complex interacts directly with core vesicle fusion machinery. For example, VPS33 binds the lipid PX domain of the vacuolar protein Syntaxin-7 that is constitutively present on yeast vacuoles (Stroupe et al., 2006) but does not exist in orthologous mammalian proteins (Nickerson et al., 2009). It is possible that as evolution progressed from yeast vacuoles to more complex lysosomes, interactions that once were constitutive became regulated by networks of GTPases such as Arl8b. Such regulation is probably critical for controlling the fusion of lysosomes with late endosomes or phagosomes through controlling the recruitment of HOPS, with consequences for processes essential to mammalian organisms such as antigen presentation and microbial defense. Interestingly, Arl8b exhibits 100% conservation of polypeptide sequence among multiple mammalian species and is among the most stably expressed genes across a variety of cell types, suggesting a strong evolutionary pressure to maintain this important molecule (Kwon et al., 2009). Furthermore, an Arl8b homolog that also mediates cargo delivery to lysosomes in *C. elegans* was identified recently (Nakae et al., 2010).

Although our data suggest Arl8b does not affect recruitment of Rab7 to lysosomes, we cannot rule out mechanisms in which Rab7 functions upstream of Arl8b in trafficking. Previous data, primarily in *S. cervisiae*, have implicated the GTPase Ypt7 (Rab7) in recruiting HOPS to late endosomes and have suggested that VPS41 functions as an effector of Rab7 (Rink et al., 2005). One model reconciling Rab7 recruitment of VPS41 with the data presented here posits that Rab7 on late endosomes recruits the HOPS complex, which in turn is recruited to lysosomes by Arl8b. In this way HOPS would directly bring late endosomes and lysosomes together for fusion, with Rab7 serving as an organizing GTPase on late endosomes and Arl8b as an organizing GTPase on lysosomes. Alternatively, Rab7 and Arl8b present on the same vesicle may work cooperatively in the recruitment of HOPS complex members in mammalian cells.

A recent report identifies Arl14 as a controller of MHC class II transport along the actin cytoskeleton (Paul et al., 2011). Together, these reports implicate the Arl family of GTPases as critical regulators of vesicle traffic and immunological host defense. Microbial infection is a constant tug-of-war between host trafficking molecules directing antigen presentation and microbial killing and the microbes themselves that seek to evade these defenses. *Mycobacterium tuberculosis*, *Legionella pneumophila*, *Salmonella enterica*, and many other microbes are known to manipulate host trafficking GTPases of the Rab and Arf families to avoid antigen presentation and microbial killing (Flannagan et al., 2009; McGhie et al., 2009). Like Rabs and Arfs, perhaps Arls may be targets of microbial immune evasion strategies. Here, we have defined an important role for Arl8b in cargo delivery to lysosomes for antigen presentation and microbial killing suggesting that Arl8b has important roles in a variety of cellular and immunological functions of lysosomes.

## Experimental Procedures

### Cell Lines and Reagents

U937 cells (ATCC) were cultured in RPMI 37°C, 5% CO2 supplemented with 10% fetal bovine serum (Hyclone), L-glutaminse (2 mM), Pen-Strep, sodium pyruvate, and β-mercaptoethanol (55 μm). The human NKT cell clones BM2a.3 and J3N.5, as well as the murine NKT hybridoma DN32, were described previously and cultured in the same medium supplemented with essential amino acids, nonessential amino acids, and HEPES (10 mM) (Brutkiewicz et al., 1995; Vincent et al., 2002). For generating monocyte-derived DCs transduced with shRNA, CD14^+^ peripheral blood monocytes from anonymous donors (MACS isolation, Miltenyi Biotec) were cultured in granulocyte macrophage-colony stimulating factor (GM-CSF) (300 U/mL) and interleukin-4 (IL-4) 200 (U/mL). RAW 264.7 macrophages (ATCC) were cultured in DMEM 37°C, 10% CO2 with the above supplements. Alexa Fluor conjugate Transferrin and Dextran were added at 10 μg/mL and .5 mg/mL in complete media unless otherwise noted. DiI-LDL was used at 10 μg/mL.

### shRNA

Please see Table S1 in Supplemental Information for a full listing of shRNA sequences.

### Lentiviral Production and Transduction

All lentivirus was produced in accordance with BL-2+ protocols publicly available at the Broad TRC website (http://www.broadinstitute.org/rnai/public/resources/protocols). VSV-G, pLKO.1, and d8.9 plasmids were obtained from the Broad RNAi Consortium. For further information, please see the Supplemental Information.

### Antigen Presentation and Cytokine ELISAS

For all assays, 50,000 antigen-presenting cells (U937, HeLa, RAW, C1R, DC)/well were plated in 96-well flat-bottom plates (Corning) with the indicated concentrations of αGal-αGalCer (Veerapen et al., 2009). After 2 hr, 50,000 responding T cells (J3N.5, BM2a.3, DN32)/well were added and incubated together at 37°C, 5% CO2. After 18–30 hr, supernatants were transferred to CoStar High Binding Plates (Cat # 3369) for sandwich ELISA. Human IFN-γ ELISAs were performed with mAb pair M700A and M701B (Thermo) and murine IL-2 ELISAs with mAb pair 554424 and 554426 (BD Biosciences). Detection was done with Streptavidin-AlkPhos (554065, BD Biosciences) and then with PNPP (N-2770, Sigma) and absorbance reading at 405 nm (Molecular Devices).

### RT-PCR and Immunoblotting

Total RNA was prepared with RNEasy and QIAshredder kits (QIAGEN) and then underwent reverse transcription with QuantiTect (QIAGEN). qPCR was performed w SYBR green (Agilent/Stratagene). See Table S2 for a complete listing of all primer sequences used in qPCR. A rabbit antisera against the C-terminal peptide of Arl8b N-TLQWLIQHSKSRRS**-**COOH was produced (YenZym). For further details, please see the Supplemental Information.

### Confocal Microscopy and Quantification

Cells were adhered overnight in complete medium to #1.5 coverslips and fixed with 3% paraformaldehyde in 1× HBSS containing Ca^2+^ and Mg^2+^ for 20 min at room termperature (RT) and then quenched with 50 mM NH_4_Cl in PBS for 10 min at RT. Permeabilization and antibody staining was done with 0.5% BSA, 0.1% Saponin, in PBS. For staining with L363 (anti-CD1d⋅αGalCer complex), a blocking step utilizing 10% goat serum was added. Cells were mounted in 15% vinol 205, 33% glycerol in PBS and analyzed on a Nikon TE2000-U inverted Microscope equipped with the laser scanning C1 confocal system. Image analysis and quantification was done with MetaMorph v7.6.4 (MDS Analytical Technologies). See Supplemental Information for further details. All scale bars represent 10 μm.

### GST Pull-downs

Please see Supplemental Information for details.

### Flow Cytometry

Cells or latex beads were stained in flow cytometry buffer (2% FBS, 0.01% sodium azide in PBS) with anti-LAMP1 at 5 μg/mL (553792 & 555798, BD), anti CD1d 5 μg/mL (553843, BD), and lastly anti-Rat PE (Invitrogen). For staining anti-CD1d⋅αGalCer (L363), 10,000 cells were first blocked with 10% normal goat serum, stained with 300 ng/mL L363, and lastly stained with goat anti-mouse PE (Invitrogen). All samples were analyzed on a FACSort flow cytometer (Beckton-Dickinson).

### Phagosomal Assays

Please see Supplemental Information for details.

## Supplemental Information

Document S1. Six Figures, Three Tables, and Supplemental Experimental Procedures
