# Supplementary material for: Lysosomal Trafficking, Antigen Presentation, and Microbial Killing Are Controlled by the Arf-like GTPase Arl8b
Source: Immunity. 2011 Aug 26;35(2):182–93. doi: 10.1016/j.immuni.2011.06.009 (PMC3584282; doi:10.1016/j.immuni.2011.06.009)
Supplement: Document S1. Six Figures, Three Tables, and Supplemental Experimental Procedures [file mmc1.pdf]

Immunity, Volume *35*

Supplemental Information

Lysosomal Trafficking, Antigen Presentation, and Microbial Killing Are Controlled by the Arf-like GTPase Arl8b

Salil Garg, Mahak Sharma, Cindy Ung, Amit Tuli, Duarte C. Barral, David L. Hava, Natacha Veerapen, Gurdyal S. Besra, Nir Hacohen, and Michael B. Brenner

Figure S1, related to Figure 1: shRNAs targeting pro-Saposin and Arl8b reduced CD1d antigen presentation

A. Sample library screening plate containing shRNAs targeting pro-Saposin and other molecules

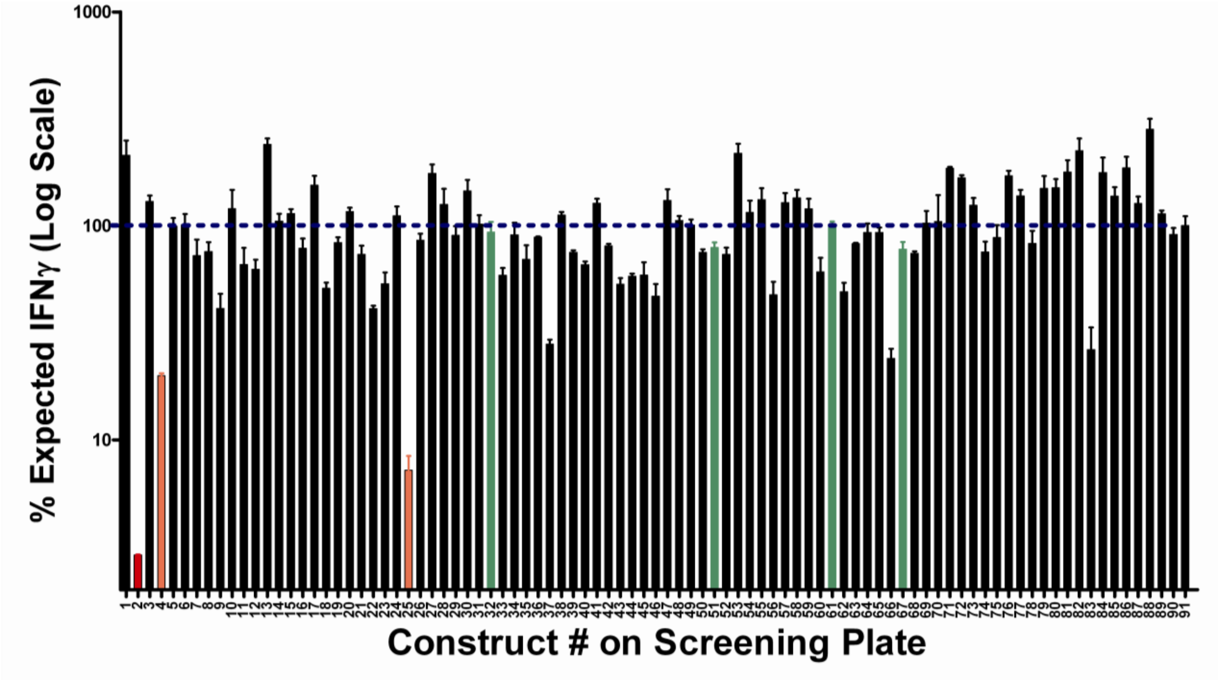

B: Multiple distinct hairpins silenced Arl8b and reduced CD1d antigen presentation

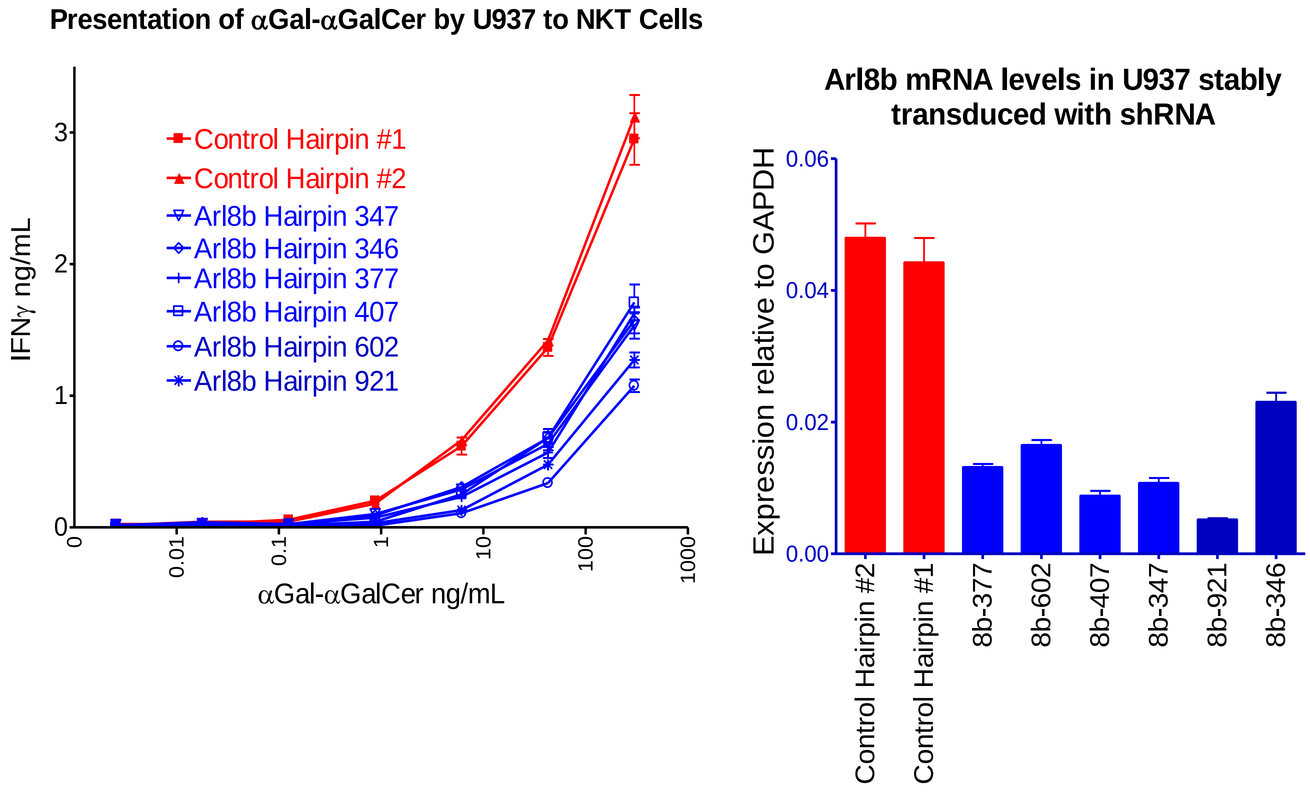

Figure S1: shRNAs targeting pro-Saposin and Arl8b reduced CD1d antigen presenation: A. Sample library screening plate containing shRNAs targeting pro-Saposin: Shown above are averages of three replicates for each shRNA construct on screening plate 15, which contained hairpins targeting pro-Saposin (red bars) amongst others. Also highlighted are negative control shRNAs randomly distributed across each screening plate targeting LacZ, GFP, and RFP (green bars). "% Expected IFN-γ" was calculated by comparing the IFN-γ secreted by NKT-cells for each construct to the best fit line for that plate (as in **Figure 1A**). "% Expected IFN-γ" of 100% indicates a shRNA which had no effect on CD1d antigen presentation. Hairpins targeting pro-Saposin (red bars) gave strong reductions in CD1d antigen presentation whereas those targeting control genes not expressed in U937 cells (green bars) did not affect IFN-γ responses. **B. Multiple distinct hairpins silenced Arl8b and reduced CD1d antigen presentation:** We generated a series of shRNA hairpins targeting various locations along the length of the mature Arl8b transcript. The numerals indicate the position of the first targeted residue from the 5' end of the mRNA molecule (see **Table 1**). U937 cells were stably transduced with lentivirus expressing each shRNA separately. *Left*: Cells were plated for antigen presentation with indicated doses of αGal-αGalCer, NKT Cells (BM2a.3) were added, and following overnight incubation response was quantified by IFN-γ ELISA. Note all hairpins targeting Arl8b gave significant reductions in dose-dependent antigen presentation compared to control hairpins. *Right*: Total RNA preps from stably transduced U937 cells were analyzed for Arl8b mRNA levels by RT-qPCR. Expression was plotted relative to GAPDH using the comparative C_t method. All six shRNA targeting Arl8b showed significant reduction in Arl8b mRNA.

Figure S2, related to Figure 2: Arl8b localized to lysosomes and controlled mCD1d entry

A. Endogenous Arl8b localized to lysosomes

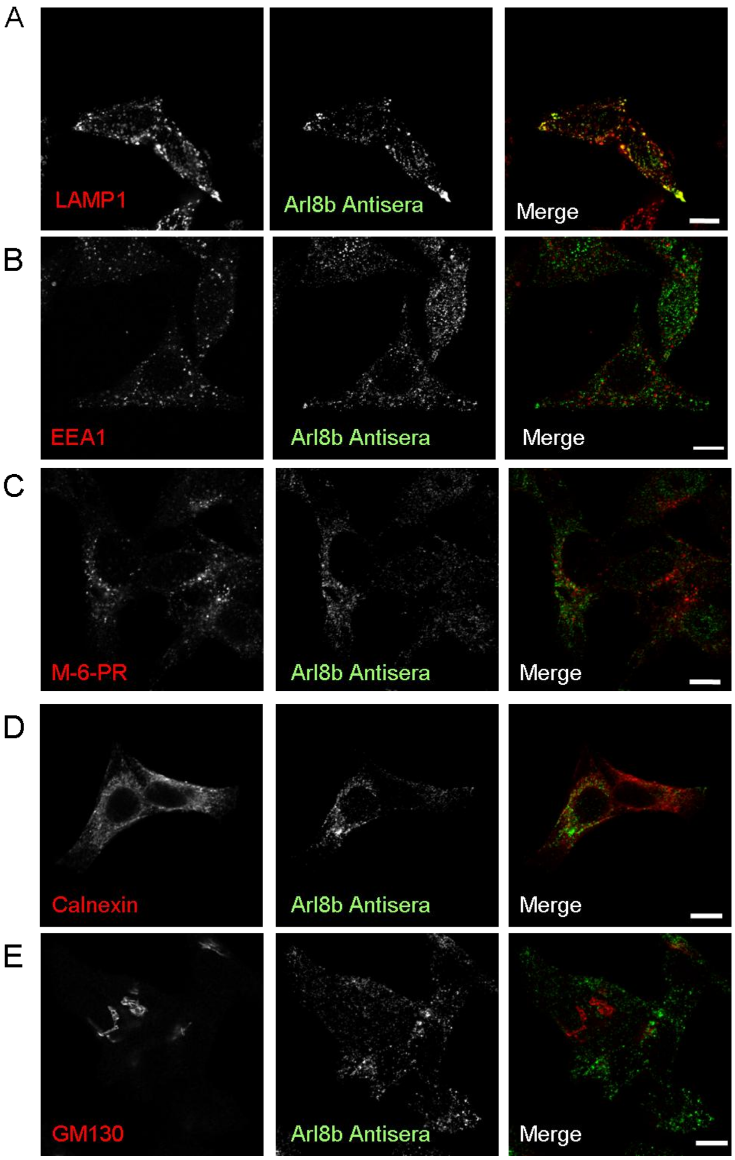

B. Quantification of Arl8b localization to intracellular compartments

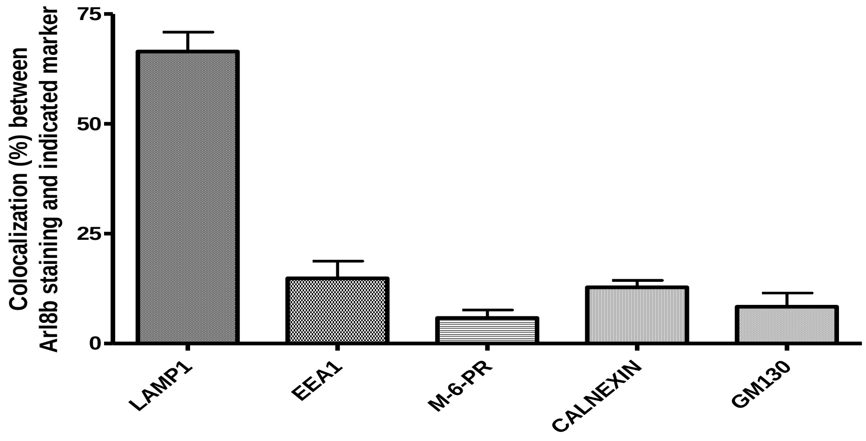

C. mCD1d was delayed entering lysosomes in Arl8b silenced cells

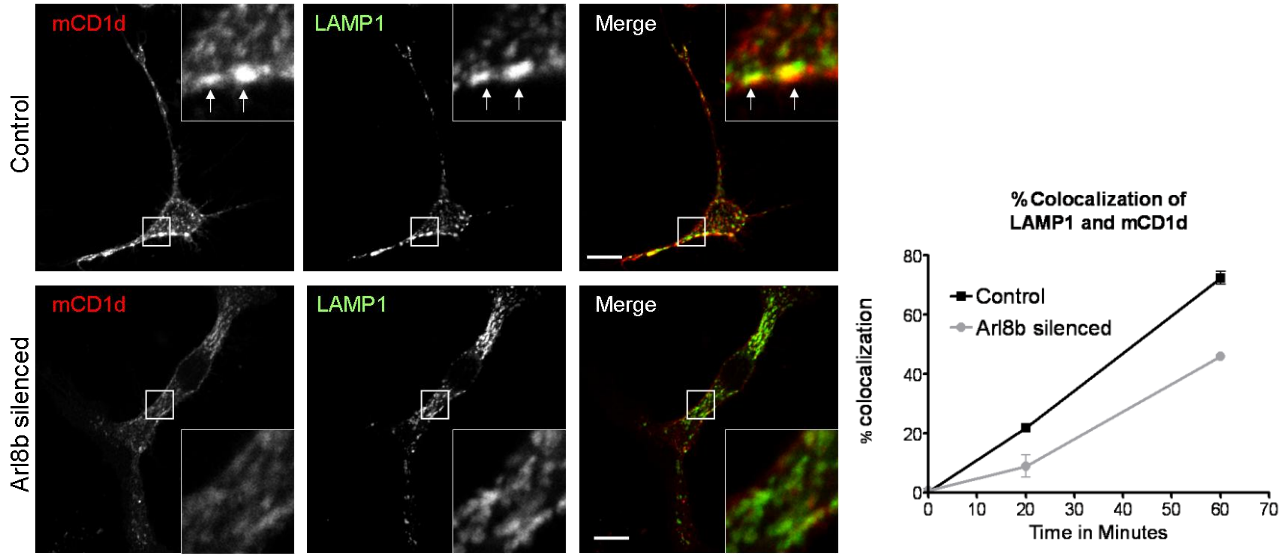

D. Steady state localization of markers to lysosomes was not impacted by Arl8b silencing

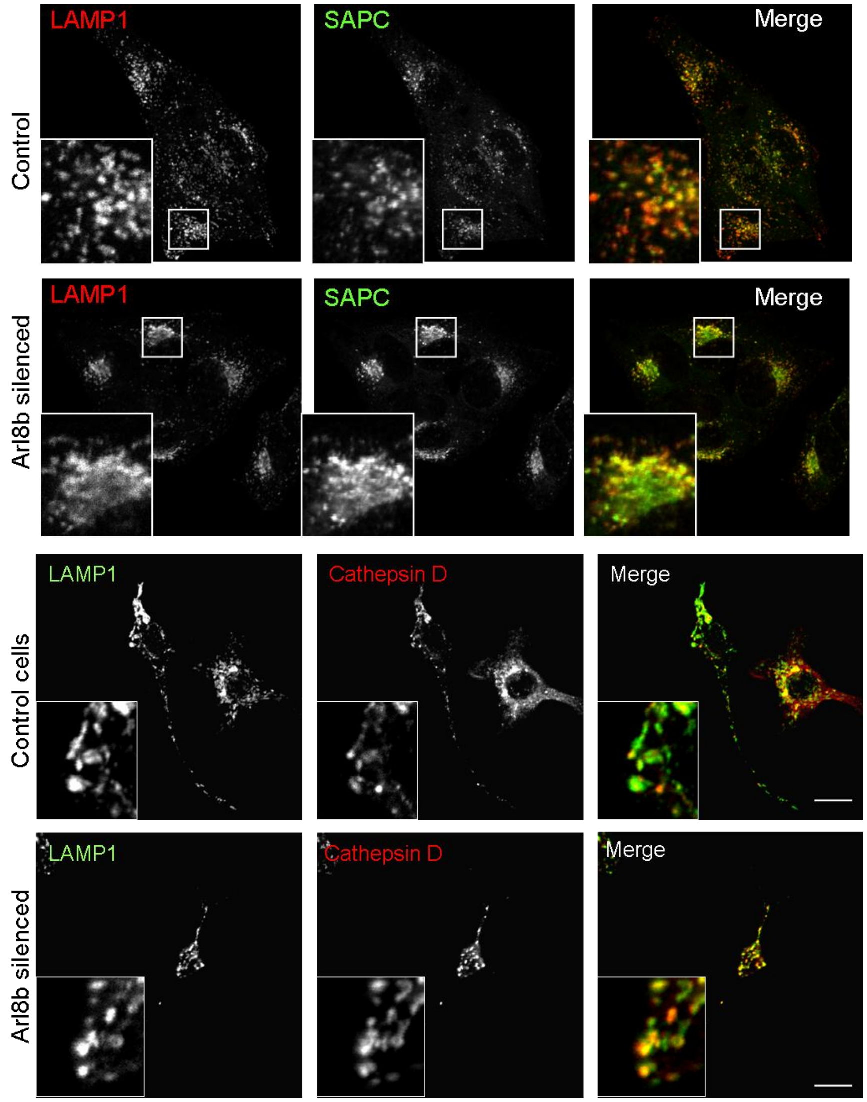

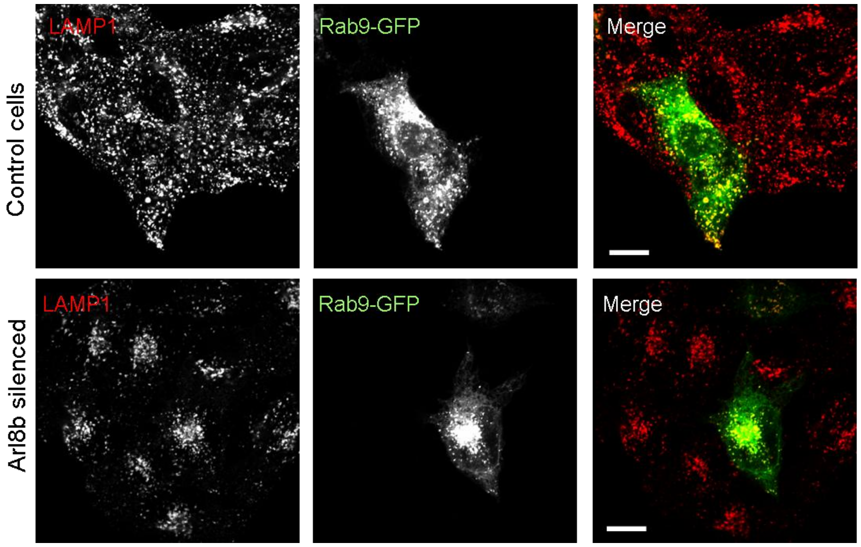

Figure S2: Arl8b localized to lysosomes and controlled mCD1d entry A. Endogenous Arl8b localizes to lysosomes: (first row) HeLa cells were stained for LAMP1 (Alexa Fluor 546, red) and with rabbit anti-Arl8 antisera followed by Alexa Fluor-488 (green) conjugated donkey anti-rabbit. The antiserum dominantly recognized LAMP1^{+} lysosomes, confirming this as the location of endogenous Arl8b. (second row) In contrast, the early endosome marker EEA1 showed no colocalization with Arl8b, indicating Arl8b did not localize to early endosomes. (third row) As in A,B, & C. M-6-PR has a complex intracellular distribution (Golgi, trans-Golgi, late endosomes, and partially to lysosomes). Arl8b did not show a significant distribution to M-6-PR^{+} compartments. (fourth row) The ER marker Calnexin showed no colocalization with Arl8b, indicating the molecule did not localize to this compartment. (fifth row) The Golgi marker GM130 showed no colocalization with Arl8b, indicating Arl8b did not localize to this compartment. Shown are representative sample images. Similar results were obtained in RAW macrophage cells. All scale bars=10μm. **B. Quantification of Arl8b localization to intracellular compartments:** Quantification of colocalization (Metamorph) between Arl8b antisera staining and the indicated markers for >30 cells for each staining in HeLa cells is shown. The pool of molecules recognized by Arl8b antisera dominantly colocalized with lysosomal marker LAMP1 and failed to colocalize significantly with markers of other compartments. **C. mCD1d was delayed entering lysosomes in Arl8b silenced cells**: mAb 19G11 was bound to CD1d in Arl8b silenced or control RAW cells at 4°C for 30 min. Cells were then warmed to 37°C for indicated times (0 minutes, 20 minutes, 60 minutes), fixed, and stained with anti-19G11 secondary (red) and anti-LAMP1 (green). (left panels) Representative images from 60 minute timepoint for Arl8b silenced (bottom row) and control cells (top row). The inset shows strong accumulation of CD1d in LAMP1^{+} lysosomes in control cells, and little or no CD1d in Lamp1^{+} compartments in Arl8b silenced cells. (right panel) Colocalization for >20 cells for each sample at each timepoint was quantified as described (methods). **D. Steady state localization of markers to lysosomes was not impacted by Arl8b silencing:** (first and second row) HeLa cells were fixed, permeabilized, and stained for saposin C (SAPC, middle panels) and LAMP1 in control (first row) and Arl8b silenced (second row) cells. Similar to control cells, Arl8b silenced cells show strong colocalization between SAPC and LAMP1. This suggests that Arl8b silencing did not result in loss of Saposin molecules from lysosomes. (third and fourth rows) RAW cells were stained for cathepsin D (middle panels) and LAMP1 in control (third row) and Arl8b silenced (fourth row) RAW cells. In both Arl8b silenced and control cells, a dominant portion of cathepsin D staining colocalizes with LAMP1. (fifth and sixth rows) Arl8b silenced (sixth row) and control (fifth row) HeLa cells were transfected with a Rab9-GFP construct, fixed, and stained for LAMP1 (red, left panels). Rab9 displays a complex distribution, labeling late endosomes, lysosomes, and trans-Golgi. Note that a portion of Rab9^{+} vesicles colocalized with LAMP1^{+} vesicles in control cells (fifth row). In Arl8b silenced cells (sixth row), a similar portion of Rab9 continued to colocalize with Arl8b. These results suggest localization of a variety of markers to lysosomes (SAP C, cathepsin D, Rab9) at steady state was unaltered in Arl8b silenced cells.

Figure S3, related to Figure 3: Arl8b amounts controlled the distribution of lysosomes

A. Arl8b silencing resulted in a mislocalization of lysosomes

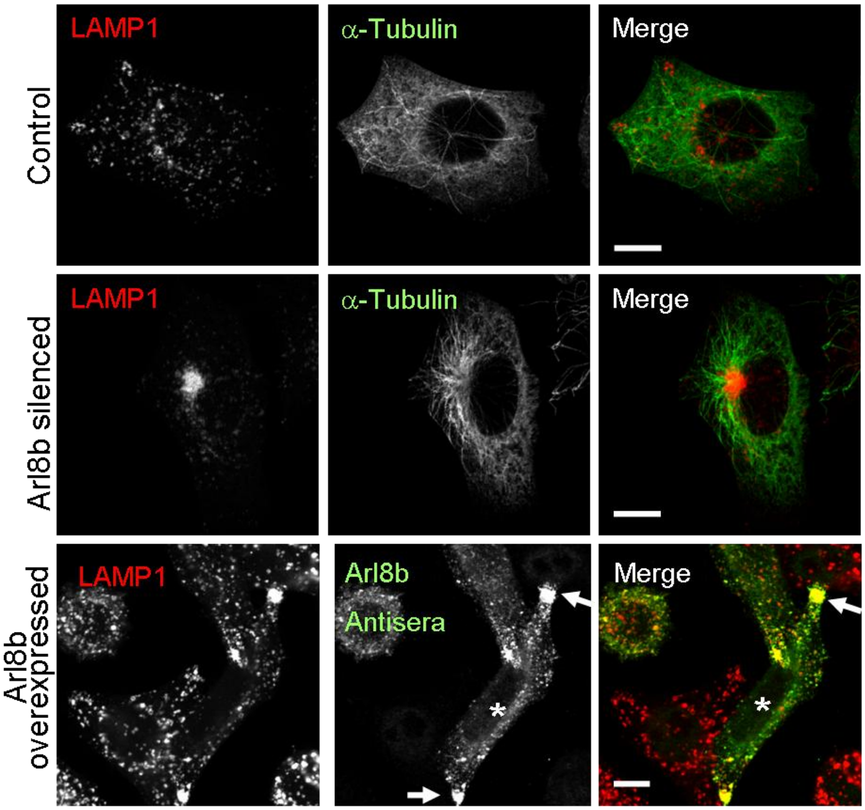

B. Arl8a and Arl8b double silencing increased clustering of lysosomes in RAW cells

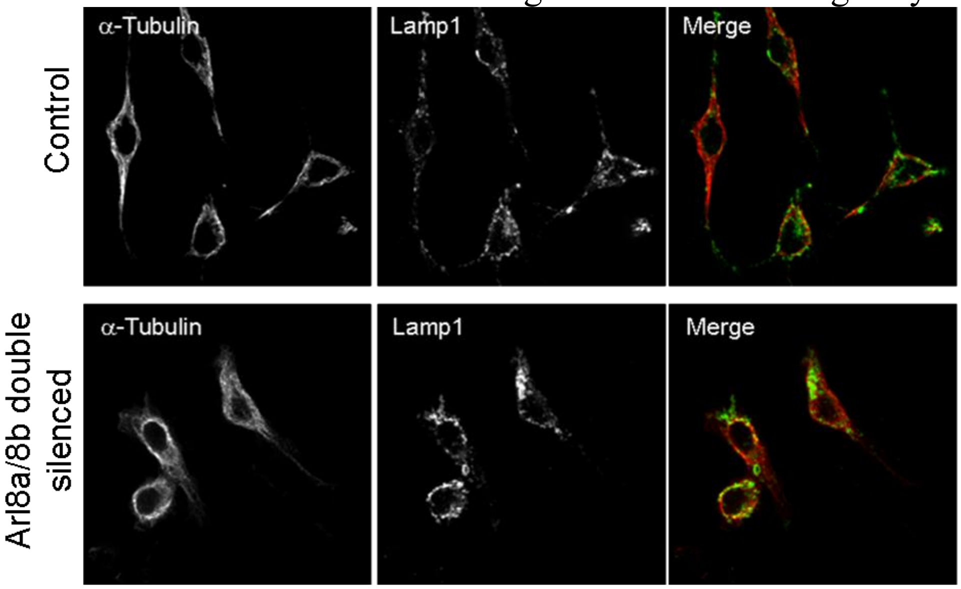

Figure S3: Arl8b amounts controlled the distribution of lysosomes A. Arl8b silencing resulted in a mislocalization of lysosomes: Arl8b silenced (middle row) or control HeLa cells (top and bottom rows) were plated on glass coverslips. (top and middle rows) Cells were fixed, permeabilized, and stained with anti-LAMP1 to mark lysosomes and anti-α-tubulin to mark the tubulin cytoskeleton and indicate the boundaries of the cell. In control cells (top row), LAMP1^{+} lysosomes distributed throughout the cytoplasm. In contrast, LAMP1^{+} lysosomes displayed a marked mislocalization to the perinuclear area of the cell upon Arl8b silencing (middle row). (bottom row) HeLa cells were transfected with an Arl8b overexpression construct, fixed, and stained with anti-LAMP1 and anti-Arl8b. An asterisk denotes a transfected cell. In contrast to Arl8b silencing, Arl8b overexpression resulted in lysosomes redistributing to the periphery of the cell and sometimes forming clusters there (indicated with arrows). **B. Arl8a and Arl8b double silencing increased clustering of lysosomes in RAW cells:** RAW cells were stably transduced with shRNA targeting Arl8b (pLKO.1 TRC1 vector, puromycin resistance) and shRNA targeting Arl8a (pLKO.1 TRC18 vector, blasticidin resistance) giving Arl8a/8b double knockdown cells (bottom row). Alternatively, RAW cells were stably transduced with two control sequences expressed from the same vectors (top row). Cells were fixed, permeabilized, and stained with anti-LAMP1 and anti-α-tubulin. Arl8 double silenced cells showed a dramatic clustering of LAMP1 when compared to control cells (compare middle panels in top and bottom row). RAW cells express both Arl8a and Arl8b and do not exhibit the same degree of lysosome mislocalization as HeLa cells when Arl8b is silenced alone (compare **A** middle row to **B** bottom row). Additionally, Arl8 double silenced RAW cells showed more dramatic lysosome clustering than Arl8b single silenced RAW cells (compare LAMP1 distribution in **B** bottom row above to LAMP1 distribution in **Supplemental Figure 2C** bottom row and **Figure 6A and 6B** bottom rows).

Figure S4, related to Figure 4: Pulldown with GST-Arl8b identified members of the HOPS complex

**Arl8b-interacting proteins**	# of unique peptides
Tubulin beta-2B chain	28
Tubulin alpha-1A chain	21
Tubulin beta-4 chain	10
Tubulin beta-6 chain	8
Tubulin beta-5 chain	4
Tubulin beta-3 chain	4
Tubulin alpha-4A chain	3
Tubulin alpha-1B chain	2
Vimentin	7
Annexin A2	5
Capza1 F-actin-capping protein subunit alpha-1	2
ATP5a1 ATP synthase subunit alpha, mitochondrial	17
ATP5b ATP synthase subunit beta, mitochondrial	17
Slc25a4 ADP/ATP translocase 1	11
V-type proton ATPase subunit H	4
**Vps16 Isoform 1 of Vacuolar protein sorting-associated protein 16 homolog**	**3**
**Vps18 of Vacuolar protein sorting-associated protein 18 homolog**	**2**
Prkaa1 protein kinase, AMP-activated, alpha 1 catalytic subunit	6
Tbc1d9b isoform 1 of TBC1 domain family member 9B	4
Iqgap1 Ras GTPase-activating-like protein IQGAP1	4

Figure S4: Pulldown with GST-Arl8b identified members of the HOPS complex: Lysates from RAW macrophages were probed with GST-Arl8b or GST. Eluates were run on SDS-PAGE. Bands which appeared specifically in the GST-Arl8b lane were cut, partially digested, and analyzed by mass spectrometry. The number of unique peptides identified corresponding to each interaction partner is listed at right. We restricted our initial analysis to those genes where at least two unique peptides were identified in this manner. Previously, Arl8b has been shown to bind β-tubulin. Encouragingly, we identified a number of tubulin subunits as potential binding partners of Arl8b. We also identified two members of the VPS-C core of the HOPS complex (see **Figures 4-5**).

Figure S5, related to Figure 5: Arl8b and VPS41 promoted the recruitment of HOPS complex members to lysosomes

A. Arl8b promoted the recruitment of VPS39 to lysosomes

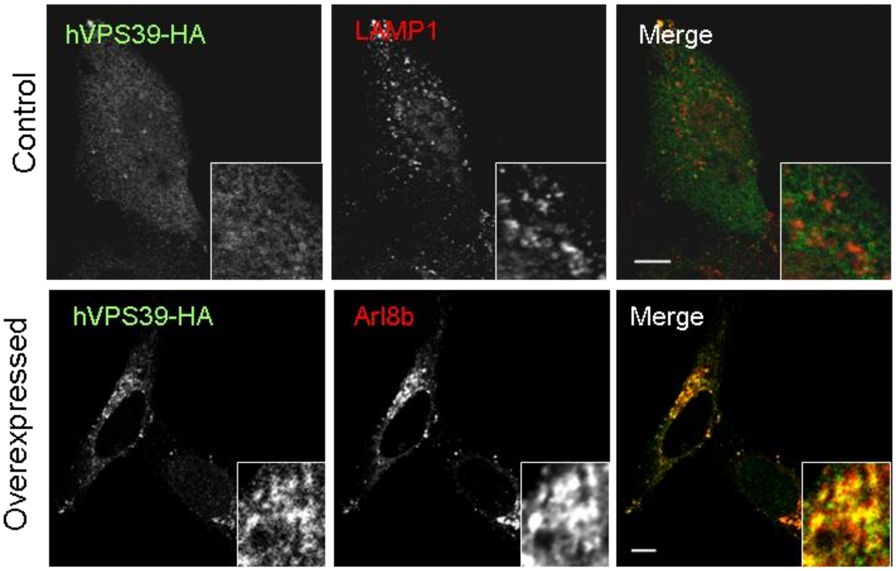

B. Arl8b and VPS41 promoted the recruitment of VPS11 to lysosomes

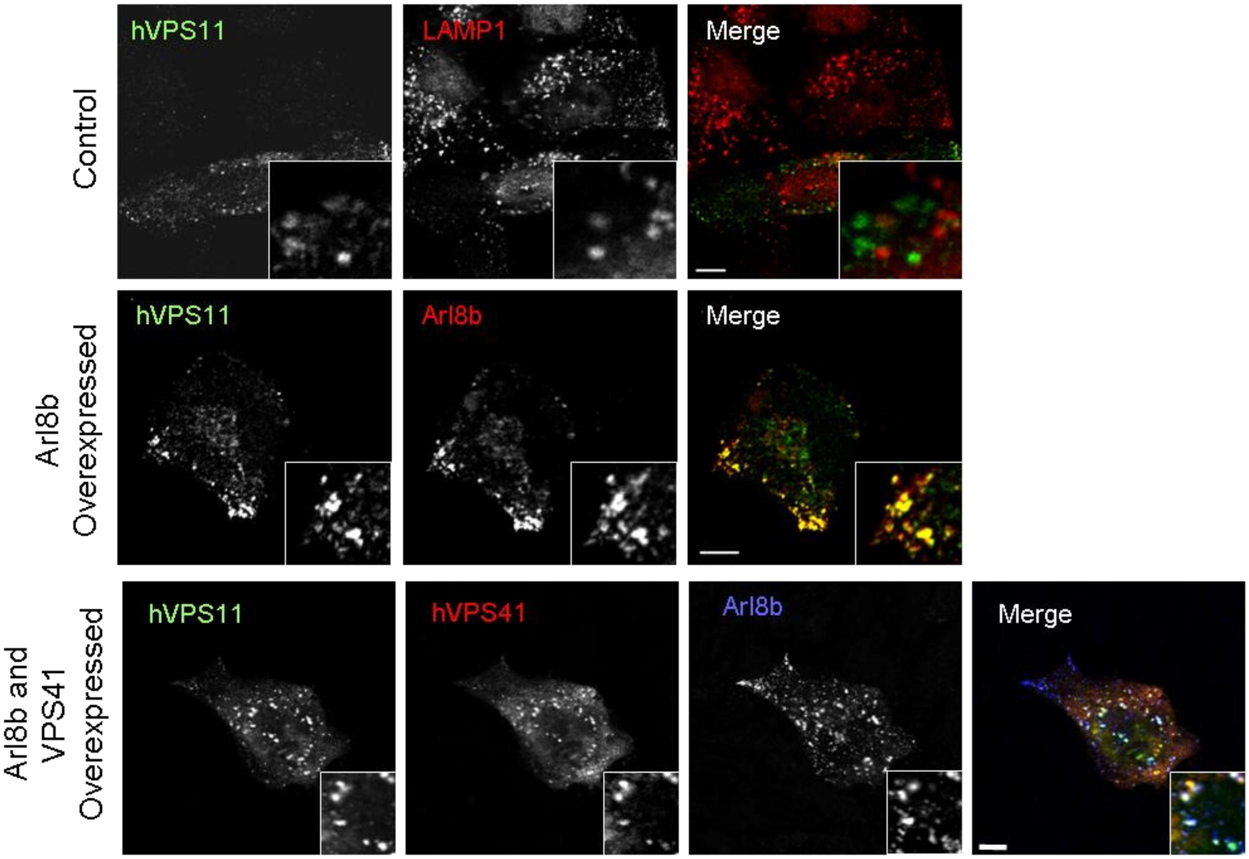

C. Arl8b and VPS41 promoted the recruitment of VPS16 to lysosomes

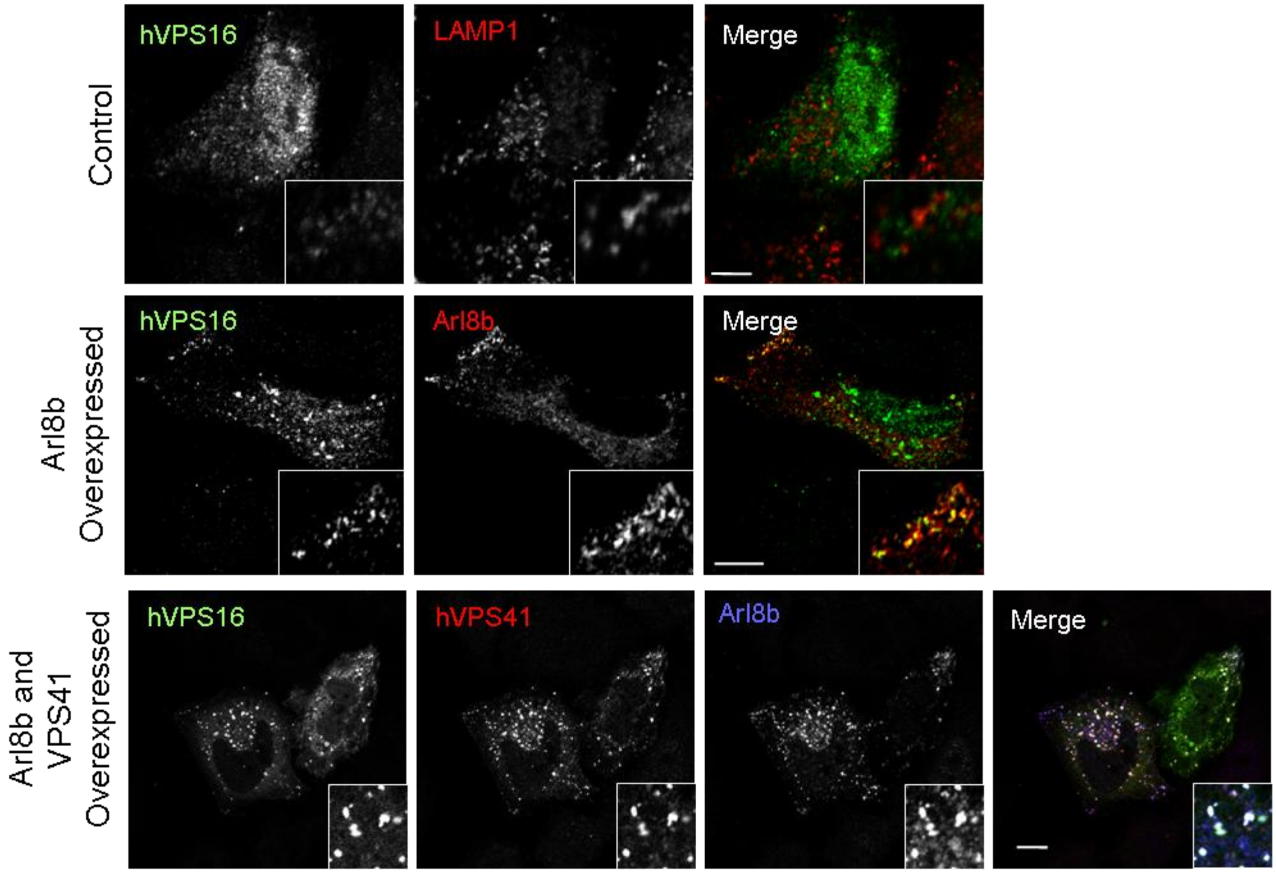

Figure S5: Arl8b and VPS41 promoted the recruitment of HOPS complex members to lysosomes A. Arl8b promoted the recruitment of VPS39 to lysosomes: HeLa cells were transfected with VPS39-HA alone or VPS39-HA with Arl8b, fixed, and stained with anti-HA (hVPS39) and anti-LAMP1. In control cells, hVPS39 distributed to the cytosol and did not colocalize with lysosomal marker LAMP1 (top row). In contrast, Arl8b overexpression resulted in a dramatic recruitment of hVPS39 to lysosomes (bottom row). **B. Arl8b and VPS41 promoted the recruitment of VPS11 to lysosomes:** HeLa cells were transfected with VPS11-GFP (top row), VPS11-GFP + Arl8b (middle row), or VPS11-GFP + Arl8b + Myc-VPS18 + VPS41-HA (bottom row). They were then fixed, permeabilized, and stained for the indicated markers. (top row) In control cells, hVPS11 exhibited a cytosolic staining pattern demonstrating little colocalization with lysosomal marker LAMP1. (middle row) Overexpression of Arl8b resulted in a partial recruitment of hVPS11 to Arl8b+ lysosomes. Note that a portion of VPS11 staining remained in Arl8b negative punctae. (bottom row) Overexpression of Arl8b, VPS41, and VPS18 resulted in near total recruitment of VPS11 to lysosomes as evidenced by the triple colocalization of VPS11, VPS41, and Arl8b staining. **C. Arl8b and VPS41 promoted the recruitment of VPS16 to lysosomes:** HeLa cells were transfected with VPS16-GFP (top row), VPS16-GFP + Arl8b (middle row), or VPS16-GFP + Arl8b + Myc-VPS18 + VPS41-HA (bottom row). They were then fixed, permeabilized, and stained for the indicated markers. (top row) Similar to VPS11 (part B above), VPS16 localized predominantly to the cytosol in control cells and did not display significant colocalization with lysosomal markers. (middle row) Overexpression of Arl8b resulted in partial recruitment of VPS16 to lysosomes. (bottom row) Overexpression of Arl8b, VPS41, and VPS18 resulted in near total recruitment of VPS16 to lysosomes as evidenced by triple staining for VPS16, VPS41, and Arl8b. Higher magnification insets are shown for clarity. All scale bars= 10 μm.

Figure S6, related to Figure 7: LAMP1 acquisition on phagocytosed beads depended on actin polymerization and occurred downstream of early endosomes

A. LAMP1 acquisition depended on actin polymerization

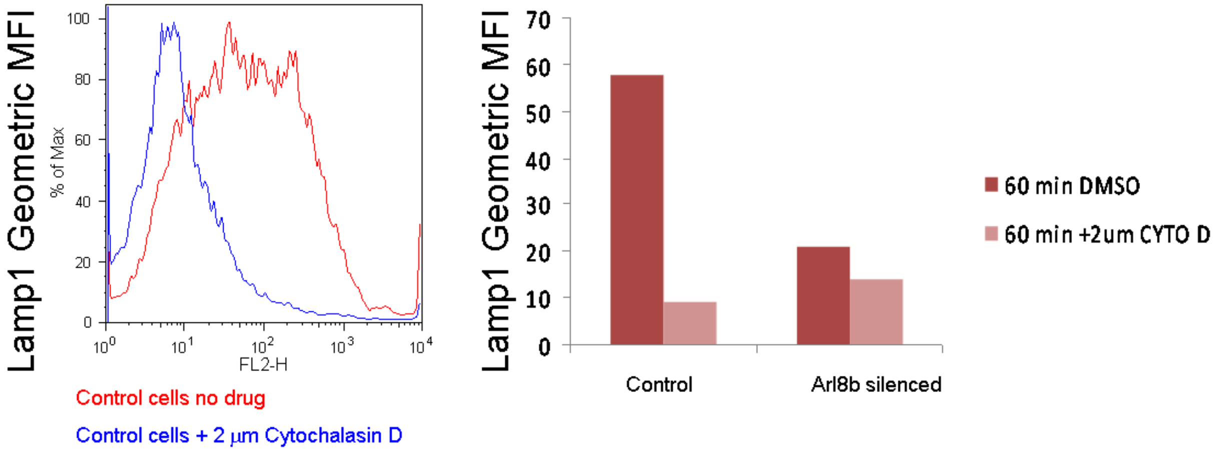

B. Phagocytosed beads isolated from Arl8b silenced cells did not show a delay in transferrin receptor acquisition

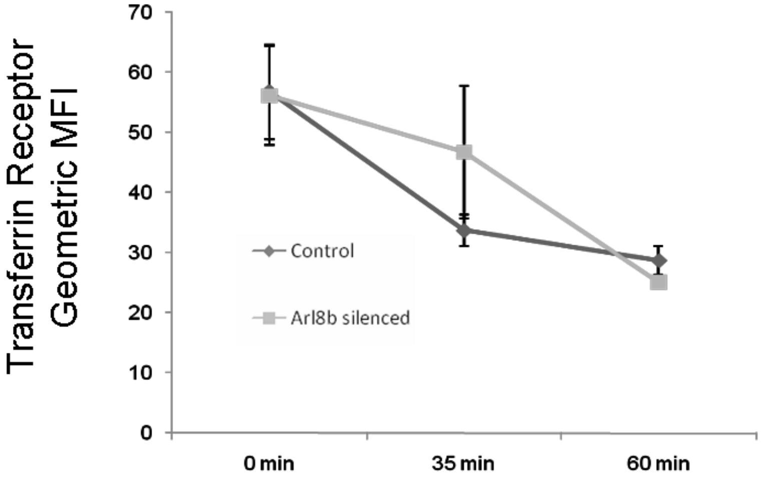

Figure S6: LAMP1 acquisition on phagocytosed beads depended on actin polymerization and occurred downstream of early endosomes A. LAMP1 acquisition depended on actin polymerization: To address the possibility that LAMP1 on phagocytosed latex beads was adsorbed non-specifically during bead repurification we treated cells with the actin polymerization inhibitor cytochalasin D. Since phagocytosis depends on actin reorganization drug treatment abolishes phagocytosis. (left panel) Pre-treatment of RAW cells with 2 μM cytochalasin D for 5 minutes resulted in a dramatic reduction of LAMP1 acquisition on latex beads following 60 minutes incubation. (right panel) The geometric MFI for LAMP1 on latex beads repurified 60 minutes after addition for both control and Arl8b silenced RAW cells is plotted with or without cytochalasin D pre-treatment. Note that in both Arl8b silenced and control cells drug treatment drastically reduced the acquisition of LAMP1 on latex beads, indicating LAMP1 acquisition depended on actin polymerization. This strongly suggests LAMP1 acquisition on beads was due to phagocytosis followed by fusion with lysosomes. **B. Phagocytosed beads isolated from Arl8b silenced cells did not show a delay in transferrin receptor acquisition.** In contrast to LAMP1, acquisition of transferrin receptor on bead phagosomes isolated from Arl8b silenced cells was not delayed compared to control cells (compare **B** to **Figure 7B**). There was a slight increase in transferrin receptor levels detected on bead phagosomes isolated 30 minutes after infection in Arl8b silenced cells, suggesting a connection between phagosomal maturation and acquisition of lysosomal markers (LAMP1) and loss of early endocytic markers (transferrin receptor).

Supplemental Experimental Procedures

Table S1: Summary of shRNA sequences utilized in this study

Name:	mRNA target:	21-mer Target Sequence:	Source:
Arl8b-407***	hArl8b, NM_018184	AGGTAACGTCACAATAAAGAT	TRC
Arl8b-346	hArl8b, NM_018184	CAGGTCAATTCAGTGAAGATA	TRC
Arl8b-921	hArl8b, NM_018184	GCTGAAGATGAATATCCCTAA	Cloned
Arl8b-377	hArl8b, NM_018184	AGTGGGCTTCAACATGAGGAA	Cloned
Arl8b-602	hArl8b, NM_018184	GCTTGGAAACAAGAGAGATCT	Cloned
Arl8b-347	hArl8b, NM_018184	AGGTCAATTCAGTGAAGATAT	Cloned
Arl8b-404	mArl8b, NM_026011	CGAGGAGTCAATGCAATTGTT	TRC
Arl8b-461**	mArl8b, NM_026011	GCCTCTCGAAATGAACTGCAT	TRC
Arl8a-1020	mArl8a, NM_026823	CCCTTTCAACACTCTGTTATT	TRC
VPS11-2265	hVPS11, NM_021729	CCTCAAGCATATCGAGAACAA	TRC
VPS16-1045	hVPS16, NM_022575	GCCAGCGAGGAAATCTTCAAA	TRC
VPS18-708	hVPS18, NM_020857	CGTGAACCGAAATGGACAGAA	TRC
VPS33A-1028	hVPS33A, NM_022916	CGAGGAAAGACACAATGCTAA	TRC
VPS33A-277	hVPS33A, NM_022916	CGGCAGCTGATGTGAAGAATA	TRC
VPS39-1883	hVPS39, NM_015289	CCTCGGCTTCTTAATAGAGAA	TRC
VPS41-2134	hVPS41, NM_014396	CCATTGACAAACCACCATTTA	TRC
MISSION*	None	CAACAAGATGAAGAGCACCAA	Sigma
GFP-437*	GFP		TRC
Annexin A1*	mAnnexinA1, NM_010730	GCTTTGGCAGATAAGTCTAAT	TRC
Annexin A6*	mAnnexinA1, NM_010730	CCTCTCTTCTTTGCTGATAAA	TRC
Empty pLKO.1*	None	N/A	TRC

All vectors listed as TRC are commercially available through Open Biosystems and Sigma. We are happy to distribute vectors cloned in the lab.

*=Each of these "control" shRNA hairpins was used in almost every experiment and found to give similar results. Additionally, results with transduction by these control shRNA were identical to non-transduced (uninfected) cells in all experiments except where both controls are shown (Figure 1b/1c). For clarity, all experiments in the manuscript utilize shMISSION transduced cells as the control unless otherwise noted.

**=Figures targeting murine Arl8b utilize this hairpin unless otherwise noted

***=Figures targeting human Arl8b utilize this hairpin unless otherwise noted

Table S2: Summary of qPCR primers utilized in this study

Target mRNA molecule:	Forward Primer	Reverse Primer
hArl8b, NM_018184	CACCTTCGTCAATGTCATCG	CCTATGTCCCAGATCTTTATTGTG
mArl8b, NM_026011	CTTCTGGAAGGAGGAGATGG	TGTCCCAGATCTTTATTGTGAC
mArl8a, NM_026823	ATTGTGTATATGGTGGATGCT	AGGGTGATGTCTATGTTGTC
hGapdh	CATTTCCTGGTATGACAACGA	GTCTACATGGCAACTGTGAG
mβ-actin	GCTCTGGCTCCTAGCACCAT	GCCACCGATCCACACCGCGT

Table S3: Summary of Antibodies utilized in this study

Target molecule:	Presumed Structures:	Manufacturer, Cat. #/Clone:
LAMP1, human	Lysosomes	BD Biosciences 555798
LAMP1, murine	Lysosomes	BD Biosciences 553792
EEA1, human	Early Endosomes	BD Biosciences 610457

EEA1, murine	Early Endosomes	Calbiochem 324610
M-6-PR	Golgi, Late Endosomes, Lysosomes	Calbiochem 444105
Saposin C	Golgi, Lysosomes	Santa-Cruz, SC-27021
mCD1d	Lysosomes, Cell surface	19G11, Gift from A. Bendelac
mCD1d	Lysosomes, Cell surface	1B1, BD Biosciences 553843
CD1d•αGalCer	Lysosomes, Cell surface	Gift from S. Porcelli
Actin (Phalloidin)	Cytoskeleton	Molecular Probes/Invitrogen
α-Tubulin	Microtubule filaments	Sigma, B512
γ-Tubulin	MTOC, Microtubule filaments	Sigma, AK-15
COPI	Median Golgi	Gift from V. Hsu
Arl8b (antisera)	Lysosomes	Raised by YenZym Antibodies
CD63	Lysosomes	BD Biosciences 556019
p150	Microtubule Filaments	BD Biosciences 610473
Anti-HA	Hemagglutinin tag	Covance, MMS-101P

Expression constructs:
Arl8b expression constructs were obtained from Origene and cloned into pcDNA3.1 using the BamH1 and EcoR1 restriction sites. Point mutants were designed and obtained using Stratagene site directed mutagenesis kits (Agilent). GFP-Rab7 and GFP-Rab9 expression constructs were obtained from Dr. Steve Caplan. GFP-RILP and GFP-ORLP1 were obtained from Dr. Jacques Neefjes. VPS expression constructs were obtained from Drs. J Wade Harper, Victor Faundez, and Robert Piper. All expression constructs were sequence verified using standard vector primers. GST-Arl8b, GST-Arl8b mutants, and GST-RILP were expressed from pGEX 4T-3 in BL-21 cells, purified using standard methods. His-Arl8b was cloned in pet15b vector using NdeI and BamH1 restriction enzymes and retransformed in BL-21 strain for protein production. Bacteria were induced with 0.5mM IPTG for 5 hours at 30 degrees C. After sonication, His-Arl8b was purified from the bacterial lysates using TALON cobalt resin (Clontech). Purified protein was eluted from the beads by using elution buffer (Clontech, HisTALON buffer set)

Lentiviral transduction:
For lentiviral transduction, U937 cells were plated in 96-well round bottom plates (10,000/well, Corning) in polybrene (8 μg/mL, SIGMA), and mixed with 10 μL of viral supernatant (Day 0). Puromycin was added after 24-48 hours at 2.5 μg/mL for a minimum of three days to select transductants. Monocyte-derived DC were transduced with lentivirus by spinoculation at 2000 rpm, 30 minutes at 25 degrees C (Day 2), puromycin added (2.0 μg/mL, Day 3) and analyzed for CD1 presentation (Day 6-7). HeLa and RAW cells were plated at 100,000/well in 6-well plates (Corning) in 5 μg/mL Polybrene and transduced by addition of 100 uL viral supernatant. 24-48 hours later, puromycin was added at 2.5 μg/mL (HeLa) and 5 μg/mL (RAW) to select transductants and experiments performed on Days 5-21 following transduction.

GST pulldowns:
GST, GST-Arl8b, and GST-Arl8b mutants were bound to GST beads. Lysates were prepared in 0.5% CHAPS buffer containing GTPγS and were incubated with purified bound GST protein overnight followed by washes with PBS or PBS containing GTPγS. Eluates were run on SDS-PAGE and subjected to electrospray ionization tandem mass spectrometry (LC-MS/MS) to discover Arl8b interacting proteins (**Supplementary Figure 4**) or were western blotted with anti-HA antibody (**Figure 4**).
His-Arl8b was first incubated with 0.5mM GTPγS in a nucleotide loading reaction. The GTP-loaded Arl8b was then incubated with Talon resin for 30 mins at 4 degrees followed by blocking of beads with

5% BSA for 1 hour. Incubation of GST proteins : GST, GST-Vps41 (Novus biologicals) or GST-RILP-Rab7 Binding domain was performed at 4 degrees C for 3 hours in 20mM Tris-HCl Ph 7.4 , 150Mm NaCl, 1mM MgCl2, 5% Glycerol , 0.5% NP-40. After the incubations, beads were pelleted by centrifugation (500g for 4 minutes at 4 degrees) followed by three washes in 25mM HEPES, 300mM NaCl, 90mM KCl, 1mM MgCl2, 0.2%Triton X-100 + 0.5mM GTPγS and then analyzed by SDS-PAGE. Western blotting was performed using anti-GST-HRP antibody (Millipore) to detect the bound proteins. Coomassie brilliant blue and Silver stain were performed to detect His-Arl8b and GST-tagged proteins respectively.

Yeast two hybrid analysis:
The *Saccharomyces cerevisiae* strain AH109 (Clontech) was maintained on Yeast extract, Peptone, Dextrose (YPD) agar plates. Transformation was carried out by the lithium acetate procedure as described in the instructions for the MATCHMAKER two-hybrid kit (Clontech). For colony growth assays, AH109 cotransformants were streaked on plates lacking leucine and tryptophan and allowed to grow at 30 degrees C for 5 days until colonies were large enough for further assays. An average of three to four colonies was then chosen and suspended in water, equilibrated to the same optical density of 600 nm and replated on plates lacking leucine and tryptophan (+HIS) as well as plates also lacking histidine (−HIS).

Arl8b antisera and western blotting
To generate Arl8b antisera, peptide was conjugated to KLH as a protein carrier, injected into rabbit, and boosted bi-weekly for 6 weeks. Rabbit IgG was then affinity purified against peptide. Lysates from 10^7 U937 or 10^7 RAW cell equivalents/lane were made in 0.5% Triton X-100 and analyzed on 15% SDS-PAGE, transferred to PVDF membranes (Bio-Rad) and blotted with Rabbit anti-Arl8b IgG (1 ug/mL) followed by donkey-anti rabbit HRP (Jackson Labs) at 1:30,000 dilution. Membranes were stripped and reprobed with anti-human β-actin (Abcam) for human cell lysates or anti-murine GAPDH (Abcam) followed by appropriate secondary HRP conjugates (Jackson Labs). All blots were developed with ECL reagents and exposed to film

Colocalization analysis using MetaMorph Imaging software:
Images from control or Arl8b silenced cells were acquired using identical laser power and gain settings on a single day within an experiment. Each channel (R,G, B) was considered separately with each pixel assigned an intensity value 0-255 with great care taken to assure that no pixel was saturated in any image used for quantification (no values = 255). A lower threshold is set whereby areas of the image not containing cells or staining are excluded, and the same threshold was always applied to control and Arl8b silenced cells. This value was 10-30 depending on the stain and never excluded visibly positive regions. The software then calculates the area overlapping between two channels where both stains are positive over the lower threshold and reports it in percentage terms. This analysis was utilized to give the reported co-localization values in **Figures 2, 5, 7**, and **Supplementary Figure 2**.

Phagosomal Assays
3 μm latex beads were purchased (Polysciences Inc) and coated by rotation with .4 mg/mL murine IgG (Sigma) for 1 hour at 37°C and added to RAW cells at a ratio of 10 beads/cell. Bead/cell mixtures were centrifuged at 300g to synchronize uptake. Following incubation for indicated times at 37°C, coverslips were washed 3x in complete DMEM to remove unbound beads, and fixed and stained as described. *E. coli* (EPEC E1348/69, gift from Lynn Bry, Harvard University) were added to RAW cells at multiplicity of infection (MOI) of 20 and centrifuged at 300g for 5 mins. The mix was then incubated for 60 mins at 37°C, 5% CO2 followed by treatment with 100 μg/mL gentamycin and chase

for the indicated times. For microscopy, cells were fixed and stained for LAMP1 and murine IgG. For CFU, cells were lysed in 0.2 % Triton in PBS for 5 mins and plated in serial dilution on LB agar plates. Following 24 hours, CFU was obtained by analysis of colonies and surviving *E. coli* per RAW cell backcalculated.
